# Targeted DamID in *C. elegans* reveals a direct role for LIN-22 and NHR-25 in antagonizing the epidermal stem cell fate

**DOI:** 10.1126/sciadv.abk3141

**Published:** 2022-02-04

**Authors:** Dimitris Katsanos, Michalis Barkoulas

**Affiliations:** Department of Life Sciences, Imperial College, London SW7 2AZ, UK.

## Abstract

Transcription factors are key players in gene networks controlling cell fate specification during development. In multicellular organisms, they display complex patterns of expression and binding to their targets, hence, tissue specificity is required in the characterization of transcription factor–target interactions. We introduce here targeted DamID (TaDa) as a method for tissue-specific transcription factor target identification in intact *Caenorhabditis elegans* animals. We use TaDa to recover targets in the epidermis for two factors, the HES1 homolog LIN-22, and the NR5A1/2 nuclear hormone receptor NHR-25. We demonstrate a direct link between LIN-22 and the Wnt signaling pathway through repression of the Frizzled receptor *lin-17*. We report a direct role for NHR-25 in promoting cell differentiation via repressing the expression of stem cell–promoting GATA factors. Our results expand our understanding of the epidermal gene network and highlight the potential of TaDa to dissect the architecture of tissue-specific gene regulatory networks.

## Teaser

LIN-22 and NHR-25 targets are identified in the epidermis of *C. elegans* by adopting Targeted DamID.

## INTRODUCTION

Development of animals and plants involves the reproducible formation of complex forms starting from single cells. This remarkable transformation requires genetic information to be decoded in a manner that allows cell type diversity to emerge through spatiotemporal control of gene expression. In this process, transcription factors (TFs) are prominent players as they participate in gene regulatory networks that govern robust cell fate specification, often acting in a combinatorial way (*1*). Therefore, elucidating the architecture of developmental gene networks by identifying the participating TFs and their biologically relevant targets is of paramount importance in the endeavor to understand how specialized cells and tissues are formed (*2*).

An important type of cells in the *Caenorhabditis elegans* epidermis are the seam cells, which follow a stem cell–like pattern of symmetric and asymmetric divisions throughout larval development to produce most epidermal nuclei (*3*). Proliferative symmetric divisions occur at the second larval stage and increase the stem cell pool, whereas asymmetric cell divisions at each larval stage generate differentiated hypodermal or neuronal cells while maintaining the total seam cell number through self-renewal. The highly reproducible nature of *C. elegans* development allows the use of this model to reveal the mechanisms underlying the balance between cell proliferation and differentiation. As in other stem cell contexts, Wnt signaling plays a key role in seam cell maintenance and the regulation of seam cell division patterns. In the canonical Wnt pathway, β-catenin is targeted for degradation in the absence of Wnt ligand binding to receptors. Upon Wnt receptor activation, β-catenin is stabilized, enters the nucleus, and along with the TF POP-1, a T cell factor/lymphoid enhancer factor ortholog, activates Wnt target genes (*4*). The Wnt/β-catenin asymmetry is a modified version of the Wnt pathway adapted for the purposes of asymmetric cell division, where asymmetric distribution of Wnt pathway components polarizes the mother cell and leads to asymmetric inheritance of the potential for Wnt pathway activation in the two daughter cells following division (*4*).

Besides Wnt signaling, a number of conserved TFs have been identified to influence cell fate decisions in the epidermis. For example, RNT-1 is the *C. elegans* Runx homolog and has been shown to act together with its binding partner BRO-1/ CBFβ to promote symmetric seam cell divisions (*5*). Mutations in Runx genes and CBFβ are known to cause various leukaemias in humans (*6*). Another example is the HES1 homolog and basic helix-loop-helix (bHLH) TF LIN-22, which acts to suppress neurogenesis in seam cells (*7*) and maintain correct patterning, possibly by antagonizing the Wnt signaling pathway (*8*). The GATA family of TFs is linked to various types of cancer in humans and related factors in *C. elegans*, such as ELT-1 and EGL-18, are thought to specify seam cell fate (*9*–*11*). Last, the NR5A1/2 homolog NHR-25 is a key example of a TF within the nuclear hormone receptor (NHR) family and regulates the establishment of cell-to-cell contacts in the seam, while it acts more broadly in vulval development, molting, and neurogenesis in the T lineage (*12*–*15*).

**Fig. 1.**
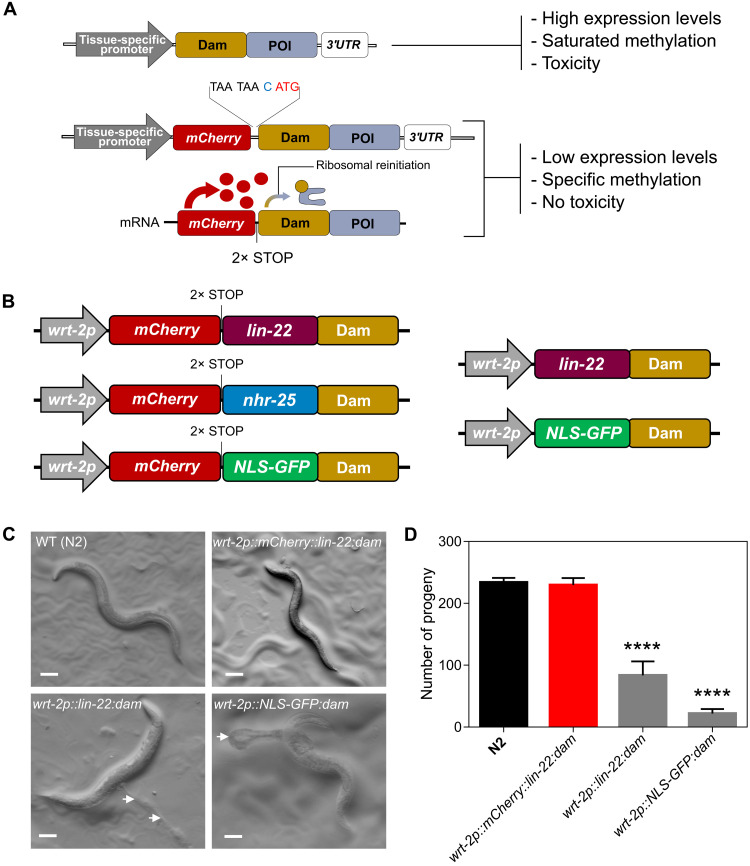
TaDa transgene design prevents animal toxicity. (**A**) Schematic showing the bicistronic design of TaDa with a primary ORF of *mCherry* followed by two STOP codons and a frameshift, which permits low levels of tissue-specific expression of a fusion between the protein of interest (POI) and Dam. (**B**) Illustration of the key features of single-copy transgenes used in this study for LIN-22 and NHR-25 target identification by TaDa (left). Transgenes used to assess the requirement of an *mCherry* primary ORF to prevent toxicity and high methylation levels are shown on the right. (**C**) Representative images of adult wild-type (WT) and transgenic animals carrying TaDa fusions. Note that animals carrying *lin-22:dam* or *NLS-GFP:dam* fusions, that is, in the absence of the primary *mCherry* ORF, show aberrant phenotypes. White arrows point to internal tissue outside the animal body. Scale bars, 100 μm. (**D**) Quantification of brood size in the above strains. Note defect in brood size in animals carrying *lin-22:dam* and *NLS-GFP:dam* fusions (*n* = 15). In (D), error bars indicate SEM, and black stars indicate statistically significant differences in the means with a one-way ANOVA followed by a Dunnett’s test (****P < 0.0001).

Despite considerable gain in knowledge on individual TFs playing a role in epidermal development, we still have a limited understanding of the underlying gene network and how this drives the decision between cell proliferation and differentiation. This is largely due to the fact that most identified components are still disconnected from each other, or when interactions are known, these remain at the genetic level. Identification of direct TF targets has been predominantly pursued by chromatin immunoprecipitation (ChIP) experiments in *C. elegans* (*16*–*18*). DNA adenine methyltransferase identification (DamID) offers an alternative way to reveal targets by fusing a TF of interest to the Dam methyltranferase from *Escherichia coli* (*19*). This enzyme mediates site-specific methylation in the adenine of GATC sequences (*20*, *21*). A key requirement in DamID methods is keeping the levels of Dam expression very low to avoid toxicity and saturated methylation of DNA (*19*, *22*). An additional challenge in multicellular systems is to characterize DNA-protein interactions with tissue-specific resolution. This has been previously achieved using recombinase-based systems to allow expression of Dam fusions in specific cells at low levels defined by the basal transcription from noninduced heat shock promoters (*22*, *23*). Targeted DamID (TaDa), first described in flies (*24*), achieves keeping low levels of Dam fusion expression by making use of a specific transgene configuration where an unrelated primary open reading frame (ORF) (usually *mCherry)* is introduced followed by two STOP codons and a frameshift preceding the Dam fusion as a second ORF (Fig. 1A). Because of the universal property of eukaryotic ribosomes to reinitiate translation after a STOP codon at a reduced frequency (*25*), this transgene design results in low levels of expression of the Dam-TF fusion in the tissue of interest as defined by the specific promoter used (Fig. 1A). This method has been used for TF target investigation in *Drosophila* to dissect mechanisms of neuronal fate determination (*26*) and in mammalian stem cell lines to characterize the binding profile of pluripotency factors (*27*).

In this study, we introduce TaDa as a powerful method to identify TF targets in *C. elegans* and use it to identify targets of LIN-22 and NHR-25 in the epidermis. Using single-molecule fluorescence in situ hybridization (smFISH) and genetic analysis, we validate changes in target expression upon perturbation of LIN-22 or NHR-25 activity. Our results suggest a role for these two TFs in promoting cell differentiation via repression of Wnt signaling and stem cell–promoting TFs. Our findings expand our knowledge of the gene network underlying epidermal cell fate decisions and provide a methodological framework toward resolving regulatory networks in specific tissues.

## RESULTS

### TaDa circumvents Dam-associated toxicity and allows the recovery of TF-specific methylation profiles

To improve our understanding of the gene network underlying epidermal cell fate patterning, we chose to study two TFs, the bHLH factor LIN-22 and the nuclear hormone receptor NHR-25. We focused on LIN-22 because previous work implicated this factor in Wnt-dependent seam cell patterning, although its direct targets remained unknown (*7*, *8*). NHR-25 is also a key player in epidermal development, and targets had been only explored at the whole-organism level using ChIP sequencing (ChIP-seq) (*14*, *16*). To profile TF targets by TaDa in the epidermis, we constructed transgenes consisting of TF-Dam fusions under the *wrt-2* promoter, which drives expression primarily in the seam cells and hypodermis during larval development (*28*). We used a *C. elegans*–optimized *mCherry* as the primary ORF, followed by two STOP codons and an extra nucleotide for frameshift before the TF-Dam (*lin-22:dam* and *nhr-25:dam)* or control (*NLS-GFP:dam*) fusions, the latter used as a reference to capture background levels of methylation (Fig. 1B). We also designed a versatile backbone to allow the assembly of N- or C-terminal fusions of any other TF with Dam under any promoter of interest (fig. S1A).

Induced ubiquitous Dam expression by a heat shock promoter has been shown to produce saturated methylation and toxicity (*19*, *20*). To test the necessity of adopting the bicistronic TaDa design, we constructed *lin-22:dam* fusions with and without *mCherry* as the primary ORF (Fig. 1B) and inserted these into the genome as single-copy transgenes. We found that transgenic lines lacking the *mCherry* primary ORF displayed developmental defects and significantly reduced brood size compared to animals containing a primary ORF (Fig. 1, C and D). Aberrant phenotypes in the absence of the primary ORF were likely due to high levels of Dam expression because they were observed in strains carrying both *lin-22:dam* and *NLS-GFP:dam* fusions and correlated with increased amount of DNA methylation (fig. S1B). We therefore conclude that the TaDa transgene configuration reduces toxicity in *C. elegans*, which is consistent with what has been reported in other systems (*19*, *24*).

We then tested the TaDa transgenes for expression and functionality of the fusion. The expression of *mCherry* was used as proxy to confirm the spatial localization of the TF-Dam fusions. Microscopy at the L4 stage revealed low *mCherry* expression in the seam cells, as expected in single copy transgenics (fig. S2A). Single copy transgenes cannot be used for rescue experiments because the TaDa design results in low level of expression of the TF-Dam fusions. However, we reasoned that functionality could be assessed using a multicopy transgenesis assay, where an increase in copy number may allow rescuing loss-of-function mutations or inducing ectopic phenotypes. For example, *lin-22(icb38)* mutants show an increase in the number of postdeirid (PDE) neurons, and this phenotype was suppressed in transgenics carrying the *lin-22:dam* construct as an extrachromosomal array and not in animals carrying *NLS-GFP:dam* fusions (fig. S2B). Furthermore, the *nhr-25:dam* fusion as a multicopy array recapitulated the increase in seam cell number that is caused by epidermal overexpression of *nhr-25*, while this phenotype was not observed in *NLS-GFP:dam* controls (fig. S2C).

We chose to profile animals at the L2 stage, when the seam cells are still proliferative, and the L4 stage when seam cell divisions are completed. To this end, we sequenced amplicons derived from methylated genomic sequences for both TFs at these two stages and processed the data using the damidseq pipeline (*29*). We generated normalized aligned read count maps to evaluate replicate reproducibility and assess the level of correlation between samples. High level of correlation was observed within TF-Dam or control sample comparisons rather than between them (fig. S3, A and B). Control *NLS-GFP:dam* samples were found to cluster separately from TF-Dam samples using principal component analysis (fig. S3C), and separate clustering was observed for the *lin-22:dam* and *nhr-25:dam* samples (fig. S3D). These results highlight that TF-Dam fusions produce specific and distinct methylation patterns compared to control fusions.

### NHR-25 and LIN-22 binding profiles overlap with regulatory regions of the genome

**Fig. 2.**
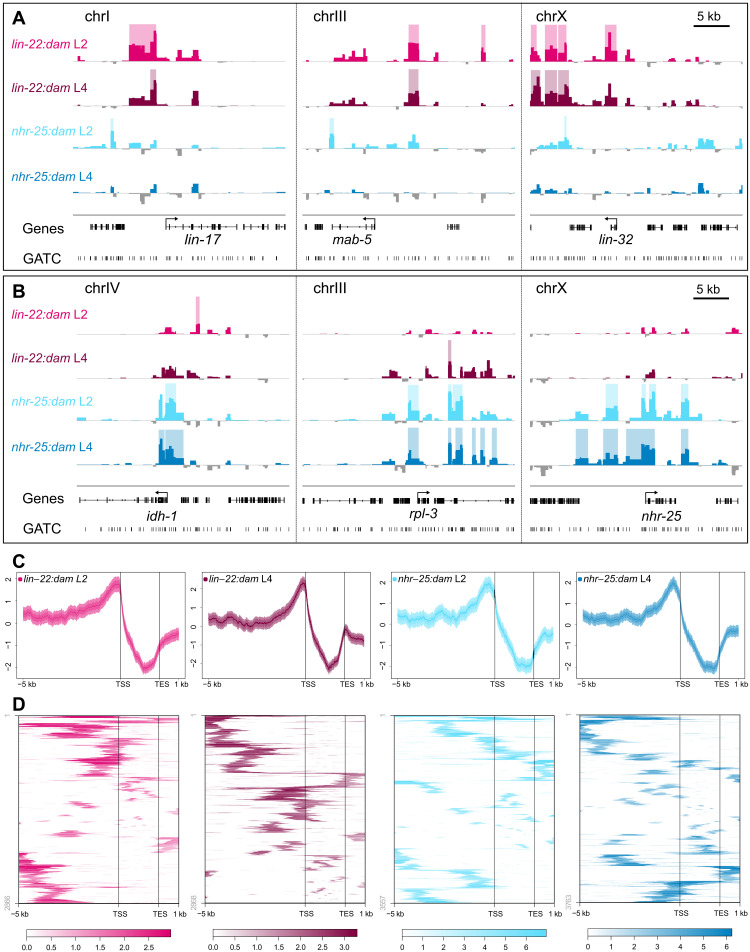
LIN-22 and NHR-25 binding signal is enriched in upstream gene regulatory regions. (**A** and **B**) Examples of signal profiles in selected regions associated with putative target genes. Shaded regions indicate statistically significant peaks (FDR < 0.05). The *y* axes represent log_2_*(TF:dam*/*NLS-GFP:dam)* scores (data range for *lin-22:dam*: −1 to 3.5 and for *nhr-25:dam*: −3 to 8). Scale bar length is 5 kb as indicated. (**C**) Aggregation plots depicting average enrichment scores in 10-bp bins for regions of equal length across all of the specified genomic features indicated on the *x* axes. Strong enrichment preference is seen upstream to gene regions. Plots show 5 kb upstream of the TSS of genes to 1 kb downstream of the TES, with gene bodies pushed into a 2-kb pseudo-length. *Y* axes are *z* scores for the plotted sequence length and shaded areas represent 95% confidence intervals. (**D**) Heatmaps representing the hierarchically clustered localization and enrichment score of all statistically significant peaks (FDR < 0.05) within 5 kb upstream and 1 kb downstream of a gene.

To investigate the profile of signal enrichment across the genome, we compared *TF:dam* and *NLS-GFP:dam* sample pairs and calculated the normalized log_2_(*TF:dam/NLS-GFP:dam*) ratio scores per GATC fragment. An example of these ratios covering 1 Mb of sequence is shown in fig. S3E. To address whether the observed signal enrichment is likely to reflect TF binding, we first focused on putative targets of LIN-22 and NHR-25 inferred by genetic analysis or ChIP-seq experiments, respectively. We observed significant enrichment in the *lin-22:dam* profiles near the Frizzled receptor *lin-17*, the Hox gene *mab-5*, and the *C. elegans achaete-scute* homolog *lin-32*, which are reported to be genetic interactors of *lin-22* (Fig. 2A) (*7*, *8*). With regard to the *nhr-25:dam* profiles, significant enrichment was found in regions near the putative target genes *idh-1* and *rpl-3,* as well as the *nhr-25* locus itself, supporting previous evidence for self-regulation (Fig. 2B) (*14*). Qualitative inspection of these enrichment profiles suggested a preference for enrichment in intergenic regions (Fig. 2, A and B). Aggregate genome-wide signal profiles showed increased average enrichment scores in upstream sequences proximal to the transcriptional start site (TSS) of protein coding genes (Fig. 2C).

**Fig. 3.**
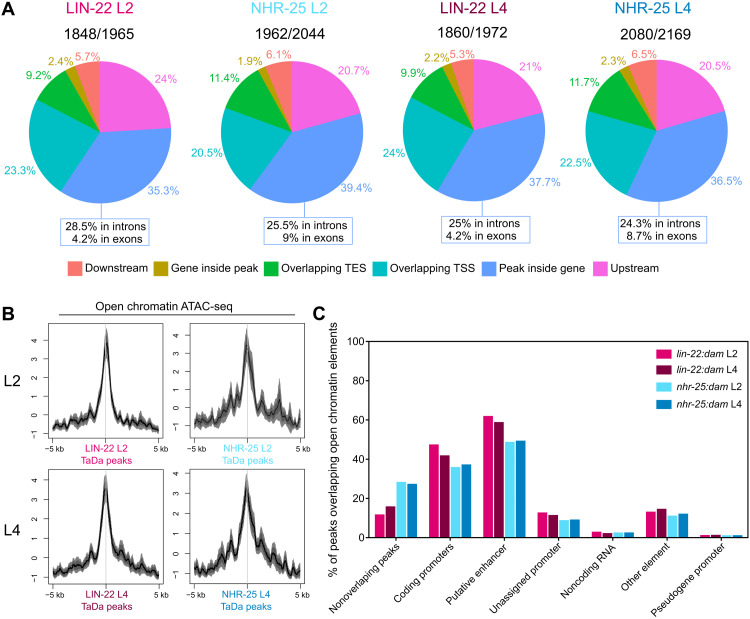
LIN-22 and NHR-25 peaks largely overlap genomic regions with regulatory potential. (**A**) Pie charts indicate the proportions of peaks residing in various genomic locations. Peaks were assigned to the single closest gene when their center coordinate was found within 6 kb upstream and 1 kb downstream of the TSS and TES, respectively, of a gene. Ratios above pie charts show the number of assigned peaks to the total number of significant peaks found. The proportions of peaks within genes with exclusive intron or exon localization are indicated under the pie charts. (**B**) Aggregation plots of open chromatin signal from ATAC-seq L2 and L4 data (*31*) over LIN-22 and NHR-25 TaDa L2 and L4 peaks, respectively. Note increased chromatin openness at the sites of peaks. *Y* axes are *z* scores for the plotted sequence length, and shaded areas represent 95% confidence intervals. The ±5 kb around the peak centers is shown. (**C**) Proportions of total peaks for each indicated TF at each stage overlapping with different categories of regulatory annotated open chromatin elements. A single TF peak may overlap with more than one open chromatin element.

Statistical processing of the signal profiles was used to identify significant peaks. We found 1965 and 1972 peaks for *lin-22:dam* at the L2 and L4 stages and 2044 and 2169 peaks for *nhr-25:dam*, a complete list of which is shown in table S1. Hierarchical clustering of the localization and score of those peaks that lie between 5 kb upstream and 1 kb downstream of genes was consistent with a preference for signal enrichment in upstream to TSS regions (Fig. 2D). The localization of the peaks in relation to genes was further dissected by assigning peaks to their single-closest gene. For all profiles, most of the peaks (>94%) were assigned to genes, with the largest proportion of peaks (between 41.2 and 47.3%) found to be upstream to or overlapping the TSS of their assigned gene (Fig. 3A). From the peaks classified as upstream of genes, approximately half (46 to 54%) localized within the first 2 kb upstream. A high proportion of peaks were found to be within genes, but around a quarter of those (24.3 to 28.5%) were exclusively residing within introns, which are known to contain regulatory elements (Fig. 3A) (*30*). In stark contrast, a very small percentage of peaks (4.2 to 9%) were found exclusively within exons. A complete list of assigned peaks can be found in table S2.

To consolidate the link between the identified peaks and putative regulatory regions of the genome, we studied the overlap between our data and open chromatin signal tracks from assays for transposase-accessible chromatin using sequencing (ATAC-seq) performed on whole-animal L2 and L4 *C. elegans* (*31*). TaDa peaks for both TF-Dam fusions showed increased average chromatin openness compared to neighboring regions (Fig. 3B). The genome-wide overlaps were highly significant (*P <* 0.0001 by Monte Carlo simulations) with most peaks overlapping either coding promoters (35.2 to 46.7%) or putative enhancers (48 to 61.2%) (Fig. 3C). We finally investigated the overlap between our data and tissue-specific open chromatin elements at the L4 stage described in a recent study (*32*). We found significant overlap with hypodermally enriched accessible chromatin (10.8 to 41.5%, *P <* 0.0001) in comparison to other tissues like neurons (3.4 to 2.4%) or the germ line (0.8 to 1.7%) where the overlap was not significant. Together, the overall localization profiles of TaDa peaks strongly support that TaDa signal is likely to reflect genuine LIN-22/NHR-25 binding sites.

### Comparisons of peak localization profiles across methods and TFs

**Fig. 4.**
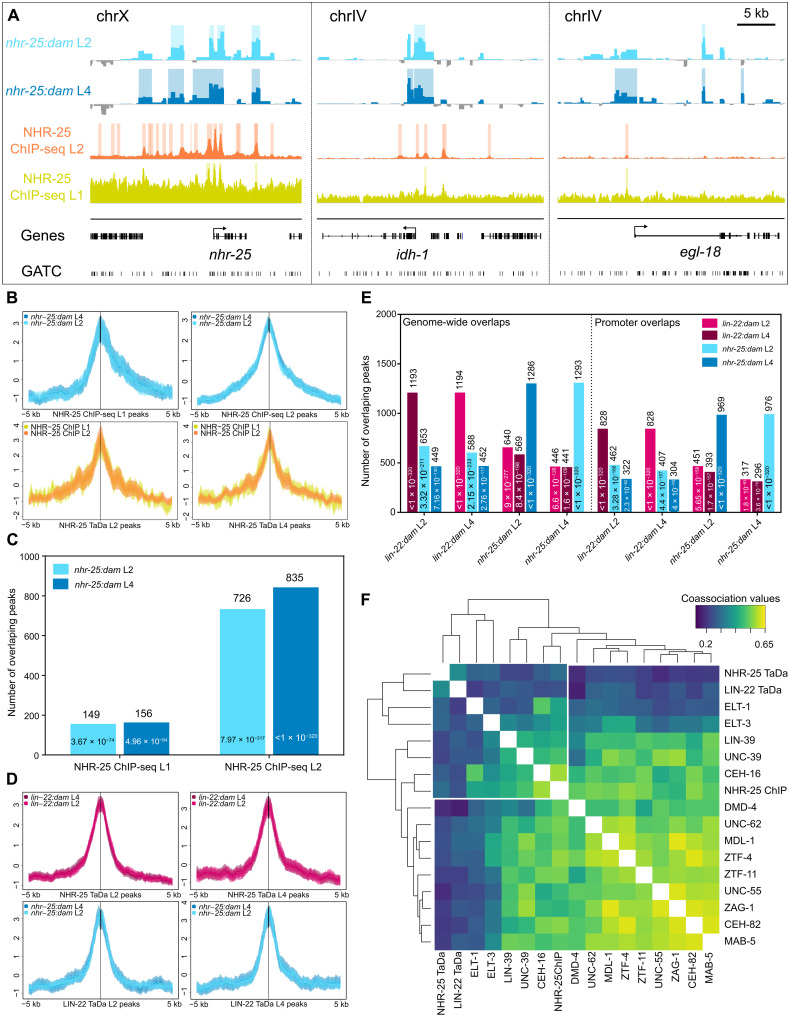
Peak localization profiles align with ChIP-seq data and reveal overlap between the two TFs. (**A**) Snapshots of *nhr-25:dam* TaDa and ChIP-seq profiles from L1 (*14*) and L2 (*16*) staged animals. Shaded regions indicate statistically significant peaks (FDR < 0.05). The *y* axes represent log_2_*(nhr-25:dam*/*NLS-GFP:dam)* scores for TaDa and fragment pile-up per million reads score for ChIP-seq. (**B**) Aggregation plots showing enrichment of *nhr-25:dam* signal over NHR-25 ChIP-seq peaks and vice versa. (**C**) Graph indicating the number of overlapping peaks between *nhr-25:dam* TaDa and NHR-25 ChIP-seq. (**D**) Aggregation plots of *lin-22:dam* signal over NHR-25 TaDa peaks and vice versa. (**E**) Graph showing the number of overlapping peaks between all sample comparisons and across the whole genome (left) or restricted to promoter regions (right). (**F**) Hierarchical clustering of symmetrized coassociation values for all pairwise comparisons between peak localization patterns. Peak profiles are taken from previous ChIP-seq experiments at the L2 stage (*17*) or our TaDa results. Note the separate clustering of TaDa peaks together with epidermal factors. In (B) and (D), *y* axes represent *z* scores for ±5 kb sequence around the peak centers, and shaded areas represent 95% confidence intervals. In (C) and (E), the exact number of peaks is shown above the bars and *P* values from Monte Carlo simulations inside the bar.

To compare our TaDa results against other methods, we used two published ChIP-seq datasets for NHR-25 binding at L1 and L2 stages from Shao *et al* (*14*) and Araya *et al.* (*16*). Initial qualitative assessment of the signal tracks showed some promising agreement (Fig. 4A). At a genome-wide level, aggregate signal of the *nhr-25:dam* L2 and L4 over all ChIP-seq L1 and L2 peaks, or vice versa, exhibited strong overlapping localization (Fig. 4B), supporting the similarity of peak profiles across methods. Both the L2 and L4 *nhr-25:dam* peaks showed significant localization overlaps by Monte Carlo simulations across the genome with the 683 peaks of the NHR-25 ChIP-seq L1 (reanalyzed in this study) and the 5980 peaks of the ChIP-seq L2 datasets (Fig. 4C). For example, we found a substantial overlap with the L2 ChIP-seq dataset, with approximately 37% (726 peaks) and 38.5% (835 peaks) of the total L2 and L4 *nhr-25:dam* peaks overlapping respectively. It is of note that in 75% of the 726 *nhr-25:dam* L2 peaks overlapping the ChIP-seq L2 peaks, the overlap included the highest enriched GATC fragment within the broader TaDa peak.

We then examined the overlap between the TaDa peak profiles identified for the two TFs. The *lin-22:dam* signal showed an increased average preference to map onto NHR-25 TaDa peaks in comparison to adjacent regions, and the same was true for *nhr-25:dam* signal on LIN-22 peaks (Fig. 4D). Since LIN-22 and NHR-25 both participate in epidermal development, it is conceivable that this similarity may reflect genuine proximity or overlap of TF binding sites. Alternatively, the shared peaks could also reflect genomic sites where promiscuous binding of multiple TFs may occur. These regions, usually referred to as high-occupancy target (HOT) regions, have been previously determined in ChIP-seq experiments (*16*). TaDa peaks showed significant (*P* < 0.0001 by Monte Carlo simulations) yet smaller overlap with HOT regions compared to ChIP-seq (8% for *nhr-25:dam* as opposed to 34% for NHR-25 ChIP-seq L2 peaks). It is of note that less than a third of the common peaks between ChIP-seq and TaDa overlapped with HOT regions. Furthermore, of the 2167 HOT regions found in L2 (*16*), 13% were overlapping with TaDa NHR-25 L2 peaks, as opposed to 83% with NHR-25 ChIP-seq L2 peaks.

To further investigate the overlap between the TaDa profiles for the two TFs, all the pairwise comparisons were made and found to be nonrandom by Monte Carlo simulations. Around 60% of peaks of each profile overlapped across the two stages for the same TF, whereas <33% overlapped across TFs (Fig. 4E, left). To avoid *P* value inflation when interrogating the entire genome while TF binding sites are expected to localize on promoters, overlaps and their statistical significance were recalculated for promoter regions only (defined here as 5 kb upstream to 500 bp downstream of TSS). Again, we found promoter overlaps to be highly significant (Fig. 4E, right) and represented most of the genome-wide overlaps (67 to 72%). In addition, less than a third (24.3 to 32.5%) of the overlaps in promoters across TF peaks occurred in HOT regions, suggesting that LIN-22 and NHR-25 may share targets during epidermal development.

To dissect the tissue specificity of the binding profile of LIN-22 and NHR-25, we compared it to peak profiles for various TFs from ChIP-seq experiments conducted at L2 from the modERN database (*17*). We included TFs known to act specifically in the epidermis, such as ELT-1 and ELT-3, along with others that play broader roles in development including neurogenesis to investigate the global peak localization pattern these TFs exhibit. Profile-wide comparisons of peak localization between factors based on overlap and proximity statistics can provide a measure of coassociation and statistically rank factors to highlight the broader similarity of their binding (*33*). Previous calculations of coassociation matrices have shown that TFs clustering together often regulate the same targets or act in the same tissue (*16*, *17*). We found that our NHR-25 and LIN-22 TaDa datasets cluster together with the epidermal ELT-1 and ELT-3 TFs, while ChIP-seq and TaDa profiles for NHR-25 did not cluster together (Fig. 4F). Since NHR-25 also acts in nonepidermal cells, which is likely to be captured in ChIP-seq but not in the TaDa profiles, this may explain the distinct positioning of the NHR-25 binding profile depending on the method via which this profile was acquired (Fig. 4F). These comparisons highlight the value of TaDa in revealing TF binding within a tissue of interest.

### LIN-22 and NHR-25 binding motif identification based on TaDa peaks

Next, we used the identified TaDa peaks to find putative motifs that are associated to TF binding. Sequences restricted to the overlap between L2 and L4 peaks for each factor were used to increase the probability that these contain binding sites. The de novo–identified motif for NHR-25 was consistent with the one previously reported by ChIP-seq (fig. S4A) (*16*, *34*). When this motif was run against a database of known motifs (*35*), it showed significant similarity to those of the *nhr-25* human ortholog NR5A1 (*P* = 3.04 × 10^−6^) and mouse ortholog *Nr5a2* (*P* = 2.35 × 10^−5^) (fig. S4B). The same analysis was carried out for LIN-22 and identified a motif that matches an E-box sequence (5′-CANNTG-3′) (fig. S4A), which matches reported motifs for the human ortholog HES1 (*36*). Comparison to known motifs showed significant similarity to that of the human HEY1 factor (*P* = 3.29 × 10^−3^) and HLH-1/*MyoD* (*P* = 2.45 × 10^−4^) (fig. S4B). Motifs for LIN-22 and NHR-25 that lied within statistically significant peaks that overlapped across the two factors had a mode distance of 277 bp at L2 and 427 bp at L4.

To assess whether the identified motifs broadly represent preference for TF binding as determined in TaDa signal profiles, aggregate signal was mapped onto motif sites. For the NHR-25 motif, the *nhr-25:dam* L2 and L4 signal showed increased average preference for regions that include the motif as opposed to neighboring sequences, while the *lin-22:dam* signal did not show any enrichment (fig. S4C). The reverse relationship was observed when the signal profiles were mapped onto the LIN-22 motif (fig. S4D). Some limited enrichment was observed for *nhr-25:dam* L2 data over the LIN-22 motif, which may be due to the fact that this motif is noisier, thus more frequent in the genome (fig. S4A).

### Characterization of LIN-22 and NHR-25 targets

To identify LIN-22 and NHR-25 putative targets, TaDa peaks were assigned to neighboring genes. Since TF binding peaks may be attributed to regulation of multiple neighboring genes, this assignment resulted in a set of 2809 genes for LIN-22 at L2 and 2833 genes at L4, and 3552 genes for NHR-25 at L2 and 3724 genes at L4 (fig. S5, A and B, and table S2). Most of the identified genes were shared between the two stages for each factor, with >63% of genes in any dataset being shared between L2 and L4 (fig. S5, A and B). LIN-22 controls aspects of epidermal development by repressing neurogenesis and influencing ray formation in males (*7*). These functions were reflected in related Gene Ontology (GO) terms for the identified target gene sets at L2 and L4 (fig. S5, C to E). In addition, the L2 dataset was found to be enriched for genes related to the Wnt signaling pathway (KEGG-adjusted *P* value of 3.28 × 10^−5^). The L2 and L4 datasets of putative NHR-25 targets were also enriched for multiple GO terms related to known NHR-25 biological functions in epidermal and neuronal patterning (*3*, *12*, *15*). For example, terms for structural constituents of the cuticle and molting cycle were found to be among the most significantly enriched (fig. S5, F to H).

We then sought to investigate how the genes identified via TaDa compare to those identified with other methods. To this end, we first compared the NHR-25 TaDa–identified targets with NHR-25 ChIP-seq datasets and found very significant overlap for all pairwise or higher-order dataset intersections (fig. S6A). For example, the overlap with the ChIP-seq L2 dataset containing 7438 genes contained 62% of L2 and 61.6% of the L4 TaDa genes. We reasoned that some of the nonoverlapping genes from ChIP-seq may reflect targets of NHR-25 outside the epidermis that are not captured by TaDa. Consistent with this hypothesis, tissue enrichment analysis for genes exclusively found by ChIP-seq showed higher enrichment for reproductive tissues compared to the epidermis (fig. S6, B and C).

As there are currently no ChIP-seq datasets available for LIN-22, we intersected the TaDa-identified genes with *C. elegans* orthologs from ChIP-seq dataset for HES1, the human homolog of *lin-22*. We found statistically significant overlap containing genes that showed enrichment for the same GO terms previously identified in the TaDa gene sets alone, indicating conservation in regulatory interactions of LIN-22/HES1 across species (fig. S6D). A significant overlap was also found when the LIN-22 TaDa target genes were intersected with a list of 52 in silico–predicted downstream genetic interactors of *lin-22* (*37*). These included not only *lin-32* and *mab-5* but also other genes known to participate in seam cell development like *rnt-1*, for which there was no prior evidence for regulation by LIN-22 (fig. S6D and table S2).

We lastly assessed the overlap between the identified targets for the two TFs. Significant overlap was found in all pairwise comparisons across factors with 37 to 46.4% of putative LIN-22 target genes overlapping NHR-25 datasets and 29.3 to 35.3% of putative NHR-25 target genes overlapping LIN-22 datasets (fig. S6E). The intersection between all TaDa datasets contained 663 genes, which showed enrichment for GO terms related to neurogenesis and development (fig. S6E). These results indicate that the functions of the two TFs during epidermal development are likely to be executed by a combination of distinct and shared target genes.

### LIN-22 and NHR-25 targets suggest a link to cell differentiation

**Fig. 5.**
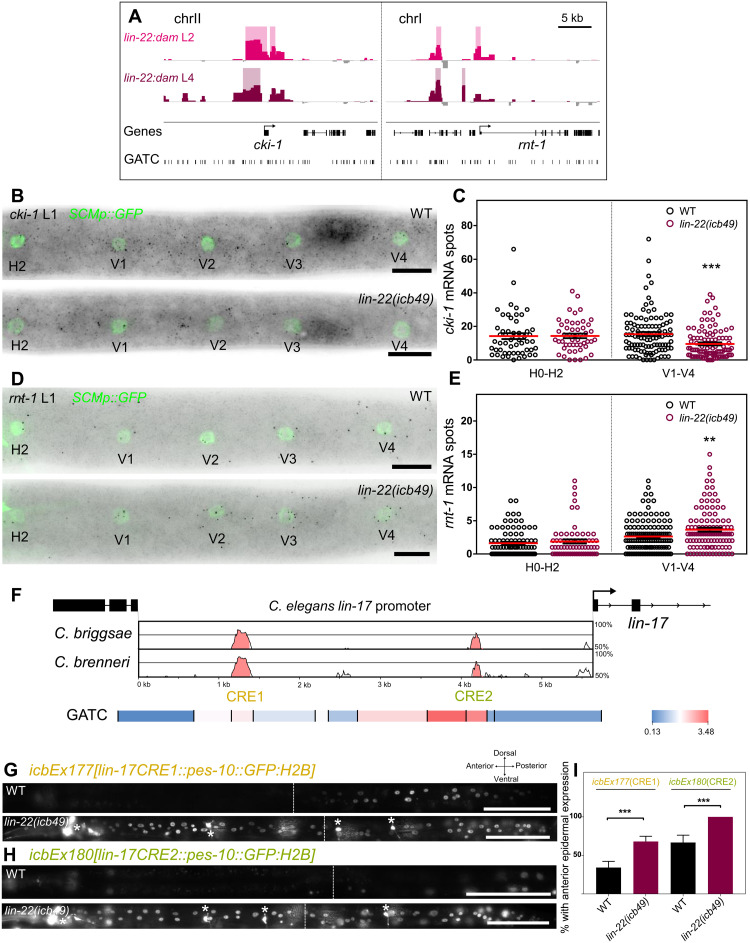
LIN-22 activates *cki-1* and represses *rnt-1 and lin-17*. (**A**) *lin-22:dam* signal around genes identified as putative LIN-22 targets. *Y* axes are log_2_*(lin-22:dam*/*NLS-GFP:dam)* scores, and shaded regions mark significant peaks. (**B**) Representative *cki-1* smFISH images at the late L1 stage. Seam cells are labeled with *SCMp:GFP*. (**C**) Quantification of *cki-1* mRNA spots in H0-V4 seam cells (50 ≤ *n* ≤ 126). (**D**) Representative *rnt-1* smFISH images at the late L1 stage. (**E**) Quantification of *rnt-1* mRNA spots in H0-V4 seam cells (65 ≤ *n* ≤ 167). (**F**) Vista analysis (*60*) of the *lin-17* promoter identified two conserved elements (*CRE1* and *CRE2*), which overlap GATC fragments with high TaDa enrichment scores (heatmap). (**G** and **H**) Representative fluorescence images of L4 animals carrying transcriptional reporters for the *lin-17 CRE1* (G) and *CRE2* sequences (H). White stars indicate *dat-1p:GFP*–expressing neurons in *lin-22(icb49)* mutants. (**I**) Quantification of the proportion of animals with epidermal expression anterior to the vulva (35 ≤ *n* ≤ 47 for *CRE1* and 20 ≤ *n* ≤ 24 for *CRE2*). Error bars indicate SEM in (C) and (E) or proportion in (I). Black stars show statistically significant differences in the means with a *t* test in (C) and (E) and a Fisher’s exact test in (I), **P < 0.01, ***P < 0.001. Scale bars, 10 μm (B and D) and 100 μm (G and H).

To validate putative interactions predicted by TaDa, we focused on selected candidates that were known to participate in the epidermal gene network, although their exact link to LIN-22/NHR-25 was unclear. With regard to LIN-22, we focused on *cki-1*, *rnt-1*, and *lin-17,* which showed significant signal enrichment in their promoter regions (Figs. 2A and 5A). First, *cki-1* encodes a cyclin-dependent kinase inhibitor protein (*38*). Comparison of *cki-1* expression between wild-type and *lin-22(icb49)* mutants revealed a significant reduction in transcript levels in V1-V4 lineages (Fig. 5, B and C). Furthermore, *cki-1* RNA interference (RNAi) treatment enhanced the increase in seam cell number observed in *lin-22* loss-of-function mutants (fig. S7A). This finding suggests that LIN-22 may activate *cki-1* expression to modulate the differentiation program.

Second, *rnt-1* is known to promote symmetric proliferative seam cell divisions (*5*). We found that *rnt-1* expression by smFISH is significantly increased in V1-V4 lineages in *lin-22(icb49)* null mutants, indicating that LIN-22 is likely to act as a repressor of *rnt-1* (Fig. 5, D and E). Consistent with this hypothesis, a loss-of-function mutation in *rnt-1* fully suppressed the increase in seam cell number observed upon loss of *lin-22* function (fig. S7B).

Third, TaDa revealed two major sites of LIN-22 signal enrichment on the *lin-17* promoter (Fig. 2A), which interestingly overlapped with two conserved regions (termed CRE1 and CRE2) between *C. elegans* and related *Caenorhabditis* species (Fig. 5F). To test the importance of these cis-regulatory elements, we constructed transcriptional reporters containing the CRE1 or CRE2 sequence fused to a minimal core promoter driving the expression of histone bound GFP. Multicopy arrays were created for each element and introduced into the *lin-22(icb49)* mutant background. Both reporters were sufficient to drive expression in the posterior epidermis in wild-type animals, while expression expanded to more anterior epidermis in the *lin-22(icb49)* mutant background (Fig. 5, G to I). This is consistent with the expansion of the *lin-17* expression domain in *lin-22* loss-of-function mutants (fig. S7C) (*8*). Together, these data indicate that LIN-22 is likely to bind to these regulatory elements on the *lin-17* promoter to repress *lin-17* expression. It is of note that LIN-22 TaDa signal was identified in the proximity of other Wnt receptors (*mom-5)* and Wnt ligands (*cwn-2*), Wnt secretion factors *(mom-1* and *mig-14*), and components of the signal transduction machinery (*lit-1* and *bar-1*) (fig. S8), indicating that LIN-22 may regulate the Wnt pathway at various levels of the signaling cascade.

**Fig. 6.**
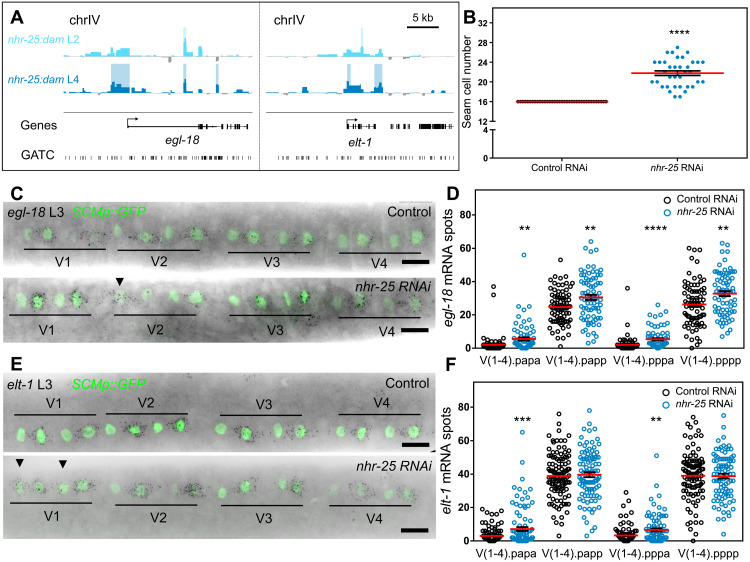
NHR-25 represses *egl-18* and *elt-1*. (**A**) *nhr-25:dam* signal forming significant peaks (shaded regions) around genes identified as putative NHR-25 targets. (**B**) Seam cell number comparison between control and *nhr-25* RNAi–treated animals used for smFISH (*n* ≥ 36). (**C**) Representative *egl-18* smFISH images of control and *nhr-25* RNAi–treated animals during the L3 division. (**D**) Quantification of *egl-18* mRNA spots in the V1-V4 daughter cells following the L3 division (60 ≤ *n* ≤ 88) showing an overall increase in expression upon *nhr-25* RNAi. (**E**) Representative *elt-1* smFISH images of control and *nhr-25* RNAi–treated animals during the L3 division. (**F**) Quantification of *elt-1* mRNA spots in the V1-V4 daughter cells following the L3 division showing a significant increase in expression in the anterior daughters (Vn.papa and Vn.pppa, 82 ≤ *n* ≤ 116). In (C) and (E), seam cells are labeled with *SCMp:GFP*, and black spots correspond to investigated mRNAs. Scale bars, 5 kb (A) and 10 μm (C and E). Arrowheads in (C) and (E) indicate instances of strong expression in anterior daughter cells. In (B), (D), and (F), error bars indicate SEM. Black stars show statistically significant differences in the mean with a *t* test, **P < 0.01, ***P < 0.001, ****P < 0.0001.

With regard to putative targets of NHR-25, we focused on *egl-18* and *elt-1*, which showed significant signal enrichment in their promoter region (Fig. 6A). These genes encode GATA TFs that promote seam cell fate and had not been previously linked to regulation by NHR-25. In particular, *egl-18* is a target of the Wnt signaling pathway, which is known to maintain seam cell fate (*9*, *10*). Expansion of the *egl-18* expression domain to the anterior seam cell daughters that normally adopt the hypodermal differentiation program has been shown to correlate with ectopic maintenance of the seam cell fate (*8*, *10*). Furthermore, *elt-1* is thought to be the master epidermal fate regulator in *C. elegans* and is known to regulate *nhr-25* in the embryo (*3*). To test whether *elt-1* and *egl-18* are likely to be targets of NHR-25, we carried out smFISH in control and *nhr-25* RNAi–treated animals at the L3 division stage. The efficacy of the RNAi treatment was confirmed phenotypically before transcript quantification (Fig. 6B). In *nhr-25* RNAi–treated animals, an overall increase in *egl-18* expression in V1-V4 daughters was observed (Fig. 6, C and D), indicating that NHR-25 is likely to be a repressor of *egl-18.* Similarly, *elt-1* expression was found to be increased in anterior daughters of the V1-V4 lineages in *nhr-25* RNAi–treated animals (Fig. 6, E and F). Knockdown of *egl-18* and *elt-1* expression by RNAi suppressed the increase in seam cell number observed in the *nhr-25(ku217)* loss-of-function mutant (fig. S7D). These findings suggest that NHR-25 may promote the differentiation program by directly repressing key seam cell fate–promoting factors.

## DISCUSSION

### LIN-22 and NHR-25 targets provide insights into epidermal cell fate specification

**Fig. 7.**
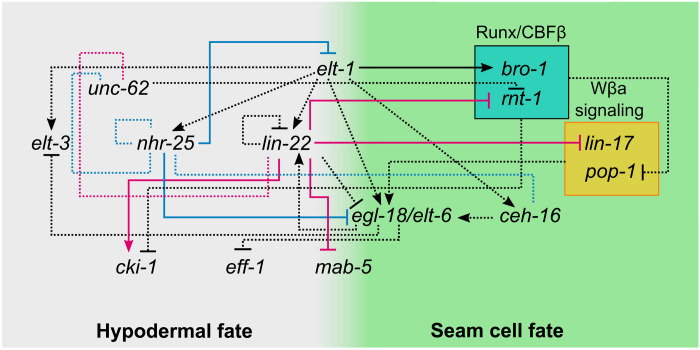
A combined gene network of epidermal cell fate interactions. Blue and magenta dashed lines indicate interactions predicted by TaDa for LIN-22 and NHR-25, respectively. Activation or repression of targets is shown on the basis of smFISH validation when known. Black dashed lines indicate published genetic interactions that are yet unknown whether they are direct or not (except for the link between *elt-1* and *bro-1*, which is likely to be direct). Hypodermal fate is shown in gray and seam cell fate in green.

Our study refines the position of *lin-22* and *nhr-25* in the epidermal gene network. In light of our TaDa findings and previous literature, we present an updated gene network and propose that LIN-22 and NHR-25 may play a prominent role in mediating cell differentiation in the epidermis (Fig. 7). For example, NHR-25 was known to influence seam cell shape and establishment of cell-to-cell contacts after cell division (*12*, *13*); however, its link to cell fate specification was not previously understood. Our findings suggest a role in the specification of the hypodermal cell fate through direct suppression of core seam cell specifying GATA TFs, such as *egl-18* and *elt-1.*

We previously proposed that LIN-22 influences seam cell divisions by antagonizing Wnt signaling through regulation of the expression of the Frizzled receptor *lin-17* (*8*). LIN-22 is also known to supress neurogenesis in V1-V4 seam cells through suppression of the Hox gene *mab-5* and the pro-neuronal factor *lin-32* (*7*). However, in all these cases, there was no prior evidence for a direct interaction, which was obtained by TaDa. Although *Hes*-related factors are commonly associated with Notch signaling (*39*), we found an unusual link between LIN-22 and Wnt signaling in *C. elegans* through potentially direct regulation of receptors and other components within this signaling pathway. LIN-22 was also found to repress *rnt-1,* the Runx homolog of *C. elegans,* which, in complex with BRO-1, promote seam cell fate and symmetric divisions by repressing *pop-1* (*5*). LIN-22 may instead activate the cell cycle inhibitor *cki-1*, which is expressed in the seam cells, and *cki-1* RNAi increases seam cell number (*38*, *40*). Genetic interactions support the possibility that increase in *rnt-1*, and reduction in *cki-1* expression may explain the supernumerary seam cells observed in *lin-22* loss-of-function mutants, although we cannot rule out that these pathways are also acting in parallel. Canonical *Hes* factors are thought to generally act as repressors (*39*); however, LIN-22 lacks a Groucho-interacting domain (*41*), so it may act as a repressor or activator depending on the availability of cofactors or competition with other TFs for binding. Beyond these genes, our TaDa lists are likely to contain many other interesting players to be studied in the future for a putative role in epidermal cell patterning.

### Expanding the *C. elegans* toolkit for TF target identification

We introduce in this study TaDa as a method to identify TF targets in a tissue-specific manner in *C. elegans*. Tissue-specific or constitutive levels of expression of Dam-fusions have been known to lead to toxicity, which we also confirmed here in the case of the *C. elegans* epidermis (*19*, *20*, *24*). The TaDa transgene configuration overcomes this obstacle by minimizing the levels of Dam expression in the tissue of interest. While methylation signal is likely to be cumulative in postmitotic cells, we show that TaDa can be used in different larval stages in dividing cell populations, such as the seam cells, leading to robust identification of putative targets.

Currently, ChIP-seq is the most commonly used method to identify TF targets in *C. elegans*, with many datasets available based on large-scale projects (*17*, *18*). We present here evidence that TaDa is comparable to ChIP-seq in identifying target genes, while it offers two key advantages. First, TaDa allows the identification of TF targets in a specific tissue of interest without cell isolation, as opposed to all tissues where the TF is expressed. Second, TaDa requires substantially less material than ChIP-seq. We found that as few as approximately 2000 animals are sufficient to allow the recovery of enough methylated DNA from the seam cells, which represent 3% of the total cells of *C. elegans*. This is also consistent with observations in mammalian cell lines, where a lot more cells were previously used to identify targets for the same TF by ChIP-seq than TaDa (*27*).

When direct comparison across methods could be made, the majority of TaDa putative target genes (>61%) were also identified by stage-matched ChIP-seq. This is very encouraging because both ChIP-seq and DamID methods have inherent biases due to the protocol and reagents used. For example, TaDa is sensitive to the quality of the Dam fusions and properties of the transgene, which may lead to off-target binding of fusions or their nonspecific expression in other tissues. The NHR-25 ChIP-seq L2 dataset was approximately three times larger in terms of peaks compared to TaDa L2 (2044 peaks in TaDa compared to 5980 in ChIP-seq). Although tissue specificity is likely to contribute to this difference, there are also alternative explanations that may decrease the size of the TaDa datasets. A key limitation in all DamID-based methods is the dependence on availability of GATC sites, which could hinder detection of some targets. However, the average length of GATC fragments in *C. elegans* is 367 bp and the median is 204 bp, so depletion of GATC sites is unlikely to be pervasive enough to substantially undermine detection of targets. TaDa peak size is reliant on the length of GATC fragments, so peaks tend to be wider and can contain multiple narrow summits of statistically significant ChIP-seq peaks. TaDa peak profiles may be distinct to ChIP-seq in other ways too, for example, they were found to be less inclusive of HOT regions, with only 13% of L2 HOT regions being represented in NHR-25 TaDa in contrast to 83% in ChIP-seq.

In summary, TaDa represents a powerful approach to dissect complex TF behaviors related to tissue-specific target binding. This includes, for example, the HLH family of TFs in *C. elegans*, which are thought to bind different targets in different tissues depending on their dimerizing partners (*42*). TaDa predictions can be further investigated with single-cell resolution using smFISH in appropriate mutant or silenced backgrounds. This experimental combination can be useful to obtain quantitative strengths of newly described interactions, thereby facilitating mathematical modeling of developmental gene networks. The revised epidermal network represents a framework for future experiments to build upon. Given the conserved nature of some of the participating factors, this network can inform more broadly about interactions that underlie robust stem cell fate patterning.

## MATERIALS AND METHODS

### *C. elegans* maintenance

The *C. elegans* strains used in this study were maintained on standard Nematode Growth Medium (NGM) plates and were grown monoxenically on a lawn of *E. coli* OP50 at 20°C. For TaDa experiments, strains were grown for at least two generations on a lawn of a *dam^−^/dcm^−^ E. coli* mutant of the K12 strain (New England Biolabs, C2925). The laboratory reference N2 strain was used as the reference strain in this study. A complete list of strains used in this study is available in table S3.

### Molecular cloning

To construct a TaDa backbone plasmid for epidermal expression of TFs fused upstream of Dam, the pCFJ151 universal *Mos1-*mediated single-copy insertion (MosSCI) vector (*43*) was digested with Bcu I/Bsp TI enzymes. The promoter of *wrt-2* was amplified by Polymerase Chain Reaction (PCR) from N2 lysate with oligos PB16 and PB7, the *C. elegans* optimized *mCherry* was amplified from the pAA64 (*34*) plasmid using oligos PB8 and PB17, the *Dam* sequence was amplified from the pUAST attB LT3 Dam plasmid (gift by T. Southall) using oligos PB18 and PB13, and the *unc-54* 3′ untranslated region (*3′UTR*) was amplified from N2 lysate using oligos PB14 and PB15. All four fragments and the digested backbone were inserted in a multifragment Gibson assembly reaction to produce the pPB7(*wrt-2p::mCherry::Dam::unc-54 3′UTR + cb-unc-119*) plasmid. The Xma JI site between *mCherry* and *Dam* was digested to linearize the vector and allow the in-frame to *Dam* insertion of the *nhr-25*–coding sequence amplified using oligos PB19 and PB20 from N2 cDNA to produce the pPB10(*wrt-2p::mCherry::nhr-25:Dam::unc-54 3′UTR + cb-unc-119*) plasmid. To construct the seam cell–driven *lin-22:Dam* fusion and the *NLS-GFP:Dam* control for the TF TaDa experiments, the *lin-22* gene was amplified with oligos DK11 and DK12 from fosmid WRM0627dG07, while *NLS-GFP* was amplified from plasmid pPD93_65 (Fire Lab vector kit, Addgene plasmid #1475) using oligos DK15 and DK16. Both amplicons were inserted upstream and in-frame with *Dam* by Gibson assembly in an Xma JI–linearized pPB7 vector like above. The resulting plasmids produced were pDK4(*wrt-2p::mCherry::lin-22:Dam::unc-54 3′UTR + cb-unc-119*) and pDK8(*wrt-2p::mCherry::NLS-GFP:Dam::unc-54 3′UTR + cb-unc-119*). To test the importance of the *mCherry* primary ORF to the viability of animals and methylation levels, versions of the *lin-22:Dam* and *NLS-GFP:Dam* TaDa constructs without *mCherry* were produced. In more detail, the pPB7 plasmid was digested with Bcu I/Mun I, and the 4085- and 6141-bp fragments were excised, extracted, and kept. The *lin-22* was amplified from pDK4 using DK102 and DK11. The two digestion fragments, *the lin-22* amplicon and the repair oligo DK103, were all inserted into a Gibson reaction to produce pDK49(*wrt-2p::lin-22:Dam::unc-54 3′UTR + cb-unc-119*), which was then digested with Bcu I/Xma JI to remove *lin-22* and inserted via Gibson assembly a DK108- and DK15-amplified fragment of NLS-GFP from pDK8 to generate pDK50(*wrt-2p::NLS-GFP:dam::unc-54 3′UTR + cb-unc-119*).

For ease of future applications, a versatile TaDa vector called pDK7 was constructed. Briefly, the *att* recombination cassette of pDest R4-R3 (Invitrogen) was amplified including the *attR4* site, *ccdb*, and *CamR* genes but excluding the *attR3* site using the oligos DK17 and DK18, including half of the *attL1* site sequence on the 3′ DK18 primer. *mCherry* was amplified from pPB7 using oligos DK19 and DK20 carrying the other half of the *attL1* site on the 5′ of DK19. *dam* was amplified from pPB7 with oligos DK21 and DK22. The *unc-54 3′UTR* was amplified from pPB7 with oligos DK23 and DK24. All four fragments were inserted in a Gibson assembly reaction along with Bcu I/Bsp TI doubly digested pCFJ151 vector to generate pDK7(*attR4-L1::mCherry::Dam-myc::unc-54 3′UTR + cb-unc-119*).

For the *lin-17 CRE1* and *CRE2* transcriptional reporters, the oligos DK115 and DK116 along with DK118 and DK119 were used to amplify each of the respective regions from N2 lysate. The Δ*pes-10* core promoter was amplified from pPD107.94 (Fire Lab vector kit, Addgene plasmid #1531) using either the *CRE1*- or the *CRE2*- compatible forward primers DK117 and DK120 along with the DK107 reverse and was cloned along with the respective CRE amplicon in a Nhe I/Xma JI–digested pDK16 to create pDK59(*lin-17CRE1:: Δpes-10::GFPo-H2B::unc-54 3′UTR + cb-unc-119*) and pDK60(*lin-17CRE2:: Δpes-10::GFPo-H2B::unc-54 3′UTR + cb-unc-119*). A complete list of the oligos used in this study is presented in table S4.

### Single-copy transgenesis

Single-copy transgenic lines were produced using the MosSCI method (*43*). Briefly, ~30 day-one adult animals of the EG6699 strain with a *Mos1* transposon insertion on chromosome II (*ttTi5605* locus) showing the uncoordinated (Unc) phenotype were injected for each transgene insertion. All the MosSCI injection mixes composed of a universal MosSCI vector (50 ng/μl) carrying the transgene of interest flanked by the *ttTi5605* left and right recombination arms along with plasmids harboring the *Mos1* transposase (pCFJ601 at 50 ng/μl), a heat-shock–inducible *peel-1* toxin (pMA122 at 10 ng/μl), and coinjection markers (pGH8 at 5 ng/μl, *myo-2::dsRed* at 2.5 ng/μl, and *myo-3::mCherry* at 5 ng/μl). After injection, animals were kept at 25°C until plates were completely starved. The heat shock treatment that follows was performed at 34°C for 3.5 hours, after which the plates were allowed to recover for 3 hours at room temperature before “reverse chunking” was performed, where NGM chunks from the lawn of a fresh plate were placed on top of the starved, treated plate with the OP50 lawn facing upward. The next day, the top of lawns were screened for *unc-119(−)* rescued animals with the absence of coinjection markers, which were transferred on different NGM plates per injected P0. Homozygous lines were confirmed molecularly for the presence of single-copy transgene insertions using oligos NM3880 and NM3884. A complete list of the transgenes produced for this study along with injection mix make-up information is available in table S5.

### Microscopy

For seam cell imaging, live animals were mounted on fresh 2% agarose pads containing 100 μM NaN_3_ for immobilization on glass slides. The slides were then imaged using either an AxioScope A1 (Zeiss) upright epifluorescence microscope with a light-emitting diode light source fitted with a RETIGA R6 camera (Q IMAGING) controlled via the Ocular software (Q IMAGING) or on an inverted Ti-eclipse fully motorized epifluorescence microscope (Nikon) with a metal halide light source fitted with an iKon M DU-934, 1024 × 1024 CCD-17291 camera (Andor) controlled via the NIS-Elements software (Nikon). Scoring of the terminal seam cell or PDE neuron number phenotype was performed in late-L4 or early adult animals, carrying the *SCMp::GFP* or *dat-1p::GFP* markers. The lateral side most proximal to the objective was counted for every animal.

To perform smFISH, large populations of animals were synchronized by bleaching and were subsequently grown at 20°C for 18 hours to reach the late L1 and 35 hours for the L3 asymmetric division stage. Animals were fixed in 4% formaldehyde (Sigma-Aldrich) in 1× phosphate-buffered saline (PBS; Ambion) for 45 min on a vertical Stuart Rotating disk (Cole-Palmer). They were washed with 1.5 ml of 1× PBS twice before being stored at 4°C in 70% ethanol for at least 24 hours for permeabilization. Hybridization was performed in 100 μl of buffer [dextran sulfate (100 mg/ml; Sigma-Aldrich) and 10% formamide in 2× SSC] at 30°C for 16 hours with 1 μl of probe diluted in water added. The probe dilution was between 1:5 and 1:50 of a custom-made mixture of 21 to 48 Cy5-labeled oligonucleotides (Biomers) targeting the gene of interest. A complete list of probes and their dilution used in this study along with their sequences is available in table S4. Animals were washed in a solution of 10% formamide, 2× SSC, stained with 4′,6-diamidino-2-phenylindole (DAPI), and resuspended for imaging in 100 μl of GLOX buffer (0.4% glucose and 10 mM tris-HCl in 2× SSC) supplemented with 1 μl of glucose oxidase (3.7 mg/ml; Sigma-Aldrich) and 1 μl of catalase (5 mg/ml; Sigma-Aldrich). Imaging was performed using the Nikon setup described above using the seam cells closest to the objective lens as homing coordinates to acquire 17 Z-stack slices with a step of 0.8 μm for each of the DAPI, Cy5, and GFP channels (Semrock). Acquisition was performed using a 100× oil immersion objective with exposure set at 100 ms for DAPI at 1/32 reduced light intensity, 3 s for Cy5, and 300 ms for GFP. Analysis was performed using a custom MATLAB (MathWorks) pipeline (*44*). Briefly, selected DAPI and GFP images were used to annotate seam cells and draw regions of interest around the nuclei for at least five slices within which smFISH spots would be counted. An animal-specific threshold for spot detection was set by manually sampling spots.

### TaDa wet laboratory protocol

Strains for TaDa experiments were transferred onto *dam^−^/dcm^−^* plates by spot bleaching, and two biological replicates in separate plates were processed simultaneously. For each strain and replicate, nine 55-mm plates fully populated by gravid adults were used for large-scale egg preparation with isolated embryos seeded in *dam^−^/dcm^−^* plates that were incubated at 20°C. Half of the resulting synchronized populations for each replicate were collected in a 15-ml centrifuge tube after 24 hours at the L2 stage and the other half after 48 hours at the L4 stage using 2 ml of M9 buffer per plate. All collected populations underwent extensive washing to remove bacteria by centrifuging animals at 1200*g* for 3 min, removing the supernatant and washing the pellet with 10 ml of M9 at least five times. Pellets were frozen at −20°C before genomic DNA (gDNA) extraction.

For gDNA extraction, pellets were lysed using 750 μl of cell lysis solution (QIAGEN) containing proteinase K (100 μg/ml) on a heat block at 55°C shaking at 500 rpm for 16 hours overnight. Lysates were treated with 4 μl of RNase A (100 mg/ml; QIAGEN) at 37°C shaking at 500 rpm for 3 hours. In turn, 250 μl of protein precipitation solution (QIAGEN) was added to each sample and was incubated on ice for 5 min followed by vigorous vortexing for at least 30 s, another 5 min incubation on ice, and a centrifugation at 6000*g* for 10 min at 4°C. The supernatant for each sample was treated with isopropanol and washed with 70% ethanol, and the pellet was air-dried for 1 hour. DNA pellets were hydrated with 55 μl of UltraPure distilled water and were left for 48 hours at 4°C for the DNA pellet to dissolve.

For isolation and amplification of the GATC-methylated gDNA fragments, the protocol followed here is an adapted version of the one presented in (*45*) for TaDa in *Drosophil*a with minor alterations. Of the extracted gDNA samples above, up to a total amount of 5 μg (range, 1 to 5 μg) was transferred into a 1.5-ml tube and was brought to 43 μl with the addition of UltraPure distilled water. For those samples where 5 μg was not available, 43 μl of the original sample was transferred. To each of those tubes, 5 μl of 10× CutSmart Buffer (New England Biolabs) and 2 μl of Dpn I restriction enzyme (New England Biolabs) were added and mixed by gentle flicking to prevent shearing of gDNA. The samples were incubated at 37°C for 16 hours overnight and were in turn cleaned-up using the QIAquick polymerase chain reaction (PCR) Purification kit (QIAGEN) and eluted with 40 μl of 50°C water. A total of 20 μl of each clean digestion product was split equally in two 0.2-ml PCR tubes (15 μl in each), and 4 μl of Adaptor ligation buffer [5× T4 DNA ligase buffer (New England Biolabs) and 10 μM of the dsAdR adaptor along with 1 μl (400 U) T4 DNA ligase (New England Biolabs)] was added in each. The samples were then incubated at 16°C for 2 hours, followed by 10 min at 65°C in a thermocycler. The double-stranded adaptor dsAdR was initially prepared by mixing equal volumes of 100 μM of the single-stranded oligos AdRT and AdRb in a 1.5-ml tube, immersing in a boiling-hot water bath, and letting it to cool down to room temperature to allow for gradual annealing. Following the adaptor ligation, each sample was mixed with 20 μl of a 2× Dpn II digestion buffer and 10 U (1 μl) of Dpn II restriction enzyme (New England Biolabs) mastermix and was incubated for 3 hours at 37°C. At this stage, for each original sample, two 40 μl of digestion reaction products were available. For methylated DNA amplification by PCR, each one of these products was mixed with 118 μl of DamID PCR buffer, and 2 μl (10 U) of MyTaq DNA polymerase (Bioline) was aliquoted at 40 μl in four different 0.2-ml PCR tubes. The DamID PCR buffer consisted of 1.36× MyTaq Buffer (Bioline) and 1.06 μM of the DamID PCR primer (Adr_PCR) that anneals on the adaptor sequence. In total, for each original gDNA sample, eight PCR reactions were performed using the following cycling program:

single cycle of steps 1 to 4: 72°C for 10 min, 94°C for 30 s, 65°C for 5 min, and 72°C for 15 min; 3 cycles of steps 5 to 7: 94°C for 30 s, 65°C for 1 min, and 72°C for 10 min; 21 cycles of steps 8 to 10: 94°C for 30 s, 65°C for 1 min, and 72°C for 2 min; final extension step: 72°C 5 min, slow cool down to room temperature and storing at 10°C.

The number of cycles for steps 8 to 10 was increased from 17 described in (*45*) to 21, which is more commonly used in previous DamID experiments in *C. elegans* (*46*). The eight reactions per sample were pooled and cleaned-up using QIAquick PCR Purification kit (QIAGEN). To remove the adaptor sequences from the resulting PCR products, up to 2.5 μg of product was transferred to a 1.5-ml tube and was digested with Alw I restriction enzyme (New England Biolabs) at 37°C for 16 hours. The products were cleaned-up again using the QIAquick PCR Purification kit. These final purified amplicons were sent to GENEWIZ for library preparation and next-generation sequencing using the Illumina HiSeq platform.

### Calculation of TaDa signal profiles for LIN-22 and NHR-25 binding

FASTQ files representing single-end reads for each sample and replicate were initially assessed using the fastq-stats perl script (available at https://github.com/owenjm/damid_misc/blob/master/fastq-stats) for uncut adaptors, primer dimer, and internal GATC content as a postsequencing quality control step for the wet laboratory executed protocol. The sequencing reads were mapped on the *C. elegans* genome, sequence alignment read-count maps were generated, and normalized log_2_(*TF:dam/NLS-GFP:dam*) ratio scores were calculated per GATC fragment of the genome using the perl script damidseq_pipeline v1.4.5 (*29*) (available at https://github.com/owenjm/damidseq_pipeline). The pipeline used Bowtie 2 v2.3.4 (*47*) for alignment to *C. elegans* bowtie indices from genome assembly WBcel235 (available from illumina iGenomes page), Samtools v1.9 (*48*) for alignment manipulations, and a GATC fragment file with the coordinates of all GATC fragments across the *C. elegans* genome in gff format, built from a WBcel235 FASTA file (available at https://ensembl.org/Caenorhabditis_elegans/Info/Index) using the gatc.track.marker.pl script (available at https://github.com/owenjm/damidseq_pipeline). The number of usable reads that have been acquired by the experiments presented here and map only once to the genome varied from over 6 million up to ~30 million reads per sample with the genome coverage being between ~9 and 45 times. From the two replicates per TF-Dam fusion and control-Dam fusion, all pairwise genome-wide log_2_(*TF:dam/NLS-GFP:dam*) calculations were performed and averaged into a single signal profile of log_2_(*TF:dam/NLS-GFP:dam*) enrichment scores per GATC of the genome for each TF at each developmental stage. Genomic coordinate files produced and used throughout this study were converted between formats (BED, GFF, Bedgraph, BigWIg, Wig, and GTF) using Excel, the Convert between GTrack/BED/WIG/bedGraph/GFF/FASTA files tool of the Galaxy powered GSuite Hyperbrowser (elixir) (at https://hyperbrowser.uio.no/hb/#!mode=advanced), and the UCSC browser binaries bedGraphToBigWig, BigWigToBedGraph, and bigWigToWig. BED, GFF, Bedgraph, and BigWig signal and feature track files were visualized using the SignalMap NimbleGen software (Roche) or IGV (*49*).

### Peak calling and gene assignment

Identification of statistically significant enriched peaks across the genome for each TF and developmental stage was performed using the perl script find_peaks (available at https://github.com/owenjm/find_peaks) with a false discovery rate (FDR) < 0.05 and default settings with the averaged log_2_(*TF:dam/NLS-GFP:dam*) signal profiles as input. The output is a list of genomic interval coordinates for statistically significant peaks with a peak enrichment score and an FDR value. Significant peaks were assigned to genes using UROPA (*50*) as a web tool (available at http://loosolab.mpi-bn.mpg.de/UROPA_GUI/) with Caenorhabditis_elegans.WBcel235.99.gtf (from http://ensembl.org/Caenorhabditis_elegans/Info/Index) as the genome annotation file. Peaks were assigned to genes on any strand when their center coordinate was positioned up to 6 kb upstream of a gene start site or 1 kb downstream of the end site. The location of the peak relative to the gene was assigned on the basis of the full length of the peak and the strand of the gene. To avoid discarding valid regulatory relationships and because of the compactness of the *C. elegans* genome, multiple assignments are reported when available.

For the NHR-25 L1 ChIP-seq dataset (*14*) used in this study for peak localization comparisons, raw sequencing data (GEO number, GSE44710) were processed into significant peak profiles using MACS2 on Galaxy (https://usegalaxy.eu/). NHR-25 and input bedgraph signal tracks were inserted into the MACS2 bdgcmp tool (default: Poisson *P* value algorithm) to deduct noise and identify NHR-25–specific signal. The output was then used with the MACS2 bdgpeakcall tool (cutoff 1.0, min-length 200 and max-gap 30) to generate genome-wide profiles of significant peaks. The total number of stringent peaks identified with this approach is smaller than the one previously reported (*14*). Profiles representing different replicates were merged using bedtools merge with averaged heights for peaks that overlap.

### Pearson’s correlation and principal component analysis

Correlation between samples and reproducibility of replicates was assessed using the deeptools3 (*51*) multiBamSummary (--binSize 300) and plotCorrelation (--corMethod pearson, --whatToPlot heatmap, --skipZeros, --removeOutliers) tools on Galaxy (https://usegalaxy.eu/). Principal component analysis was performed using the deeptools3 plotPCA tool on the multiBamSummary-calculated read count density summary matrices.

### Aggregation plots and heatmaps of signal localization

Signal localization preference around given genomic features presented as aggregation plots or heatmaps were generated using the SeqPlots GUI application (*52*) with specific settings mentioned individually for each presented result. Aggregation plots represent signal averages for 10-bp bins in regions of varying but specified length around positional features of the genome. For genes, all of their start and end coordinates, based on the largest transcript and used here as the TSS and transcriptional end site (TES) of genes, are anchored to two positions of the *x* axis, and their genic sequence is pushed or stretched to a pseudo-length of usually 2 kb. For other features, the midpoint coordinate is used to align all to the same position on the *x* axis, which then extends upstream and/or downstream of that region. For each position around the feature, an average is calculated across all the features to generate the aggregation plot line with a shaded area representing the 95% confidence interval. When *z* scores are presented on the *y* axes, those have been calculated as deviations from the mean signal seen across the plotted region. In heatmaps, each line represents each occurrence of a genomic feature indicated on the *x* axis along with a surrounding region of a given length also indicated on the *x* axis. Color scales indicate the positional enrichment score calculated as averages per 10-bp bins.

### Assessment of overlaps between sets of genomic intervals or gene sets

To identify overlapping peaks between samples or other genomic interval or features, the bedtools intersect tool was used with settings dependent on the prospected outcome of the processing. To test whether sets of genomic coordinates representing various features show statistically significant overlaps across the genome, Monte Carlo simulations have been performed using the python pipeline OLOGRAM, part of the gtftk package (*53*). *P* values are calculated on the basis of the occurrence of intersections between intervals and overall length of overlap (in base pairs) across the genome. For statistical assessment of the level of association between patterns of peak (TaDa or ChIP-seq peaks) localization across the genome for different TFs, the IntervalStats tool (*33*) as part of the coloc-stats webserver (https://hyperbrowser.uio.no/coloc-stats/) was used. Briefly, for the TFs used in this study, ChIP-seq optimal Irreproducible discovery rate (IDR)–thresholded peak coordinate files from L2 animals were downloaded from the modERN (*17*) and modENCODE (*18*) databases and were combined along with the L2 TaDa TF samples into a Gsuite of genomic tracks on coloc-stats. Each peak file was then used as query against the GSuite of reference sequences to calculate the IntervalStats statistic for colocalization for all pairwise comparisons of peak coordinates. The values in the resulting comparison matrix representing comparisons between the same two TFs with different directionality (query-reference) were averaged to symmetrize the matrix and calculate the final coassociation values that were plotted as a heatmap using the R package heatmap3 and hierarchical clustering. To assess the statistical significance of overlaps between sets of genes, hypergeometric distribution or Fisher’s exact tests were performed either on http://nemates.org/MA/progs/overlap_stats.html or using the R software package *SuperExactTest* (*54*), respectively. For both tests, when sets of coding genes are compared, the size of the sampling pool was set to 20191, the number of annotated coding genes in the WBcel235.99 release. Representation of overlaps was either in the form of Venn diagrams generated using http://bioinformatics.psb.ugent.be/webtools/Venn/ or in the form of the output of the *SuperExactTest* package.

### TF motif identification by TaDa

Identification of motifs from TF TaDa peaks was done here using HOMER (*55*). The top 200 peaks with the highest averaged enrichment score were used for the analysis. Peak interval files were used as input for the findMotifsGenome.pl script using the ce11 genome assembly, masking of the sequences, and the option to analyze the size of sequences provided by the interval file (options: ce11 -size given -mask). The logos presented here for motifs identified using homer were generated after converting the homer positional weight matrix into a transfac matrix using the RSAT (*56*) Metazoa convert matrix tool (http://rsat.sb-roscoff.fr/convert-matrix_form.cgi) and importing to Weblogo3 (https://weblogo.berkeley.edu/logo.cgi) for logo drawing. Identification of similar known motifs to the de novo identified motifs was performed using the Tomtom tool of MEME-suite (*57*). The default parameters were used, and the interrogated motif matrices were compared against the JASPAR core 2018 nonredundant database.

### Gene set enrichment analysis

Gene sets identified in this study were assessed for enriched GO terms or association with tissue-specific expression using the wormbase.org Enrichment Analysis tool (*58*, *59*) (https://wormbase.org/tools/enrichment/tea/tea.cgi), using a *q* value threshold of 0.1. For significant GO terms presented here, the −log_10_*q* value is plotted. Association of gene sets with biological pathways was evaluated using the gProfiler gOst tool (https://biit.cs.ut.ee/gprofiler/gost), and a g:SCS calculated a significance threshold of 0.05.

### RNA interference

Knockdown of *nhr-25*, *cki-1*, *egl-18*, and *elt-1* was performed by feeding animals on lawns of bacteria expressing double-stranded RNA(dsRNA) targeting each of these genes (Source Bioscience). Bacteria were grown overnight and then seeded directly onto NGM plates containing 1 μM isopropyl-β-d-thiogalactopyranoside, ampicillin (25 μg/ml), and tetracycline (6.25 μg/ml). Five L4 animals of the strain of interest were transferred on RNAi plates and were allowed to lay progeny that were phenotyped at the stage of interest. Control treatments were performed in parallel, with precisely the same experimental conditions, by feeding animals on lawns of the same strain of HT115 bacteria containing an empty dsRNA-expressing vector.

### Statistical analysis

Statistical analysis for comparisons between datasets that is not covered in the above paragraphs was performed using GraphPad prism 7 (www.graphpad.com). To test differences in the mean between seam cell scoring or smFISH counting datasets, an unpaired two-tailed *t* test was performed when the comparison was between two datasets and a one-way analysis of variance (ANOVA) was performed when multiple datasets were compared. One-way ANOVA was followed by a Dunnett’s post hoc test when the mean of multiple datasets was compared to that of a control. The significance threshold used throughout is *P* < 0.05.

## Notes

View/request a protocol for this paper from *Bio-protocol*.
