# Supplementary material for: Targeted DamID in C. elegans reveals a direct role for LIN-22 and NHR-25 in antagonizing the epidermal stem cell fate
Source: Sci Adv. 2022 Feb 4;8(5):eabk3141. doi: 10.1126/sciadv.abk3141 (PMC8816332; doi:10.1126/sciadv.abk3141)
Supplement: Supplementary file 1 — Figs. S1 to S8 Legends for tables S1 to S5 [file sciadv.abk3141_sm.pdf]

Science Advances
AAAS

Supplementary Materials for

Targeted DamID in *C. elegans* reveals a direct role for LIN-22 and NHR-25 in antagonizing the epidermal stem cell fate

Dimitris Katsanos and Michalis Barkoulas*

*Corresponding author. Email: m.barkoulas@imperial.ac.uk

Published 4 February 2022, *Sci. Adv.* **8**, eabk3141 (2022)
DOI: 10.1126/sciadv.abk3141

The PDF file includes:

Figs. S1 to S8
Legends for tables S1 to S5

Other Supplementary Material for this manuscript includes the following:

Tables S1 to S5

Fig. S1.

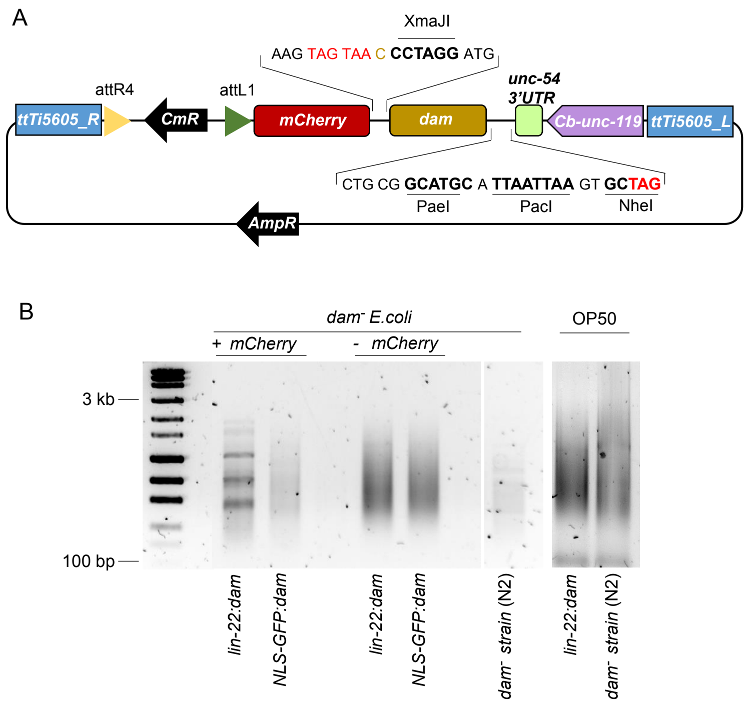

Figure S1: Assembly and assessment of *in vivo* methylation by TaDa fusions.

(A) Graphic illustration of the design of a versatile *C. elegans* universal TaDa cloning platform with its key features. From left to right the plasmid contains: universal MosSCI recombination sites (ttTi5605_R and L), an LR attR4-attL1 Gateway cloning site for promoter insertion, an *mCherry* primary ORF followed by 2 x STOP codons, indicated in red, a frameshift in yellow and a unique XmaJI restriction site followed by *dam*, unique restriction cites for PaeI, PacI, NheI and an in-frame STOP codon prior to an *unc-54 3'UTR*. **(B)** Example of amplification products from methylated gDNA extracted from strains carrying the *dam* fusions described in this study and a WT dam⁻ strain fed on dam⁻ *E. coli*. Note that all samples except for the *dam*⁻ strain show a pronounced 2 kb to 200 bp smear. Wells for all samples were loaded with the same volume of reaction product. Also note the marked increase in methylation observed in the absence of *mCherry*

as a primary ORF. Strains grown on *dam*$^{+}$ OP50 also show extensive amplification due to methylated bacterial DNA.

Fig. S2.

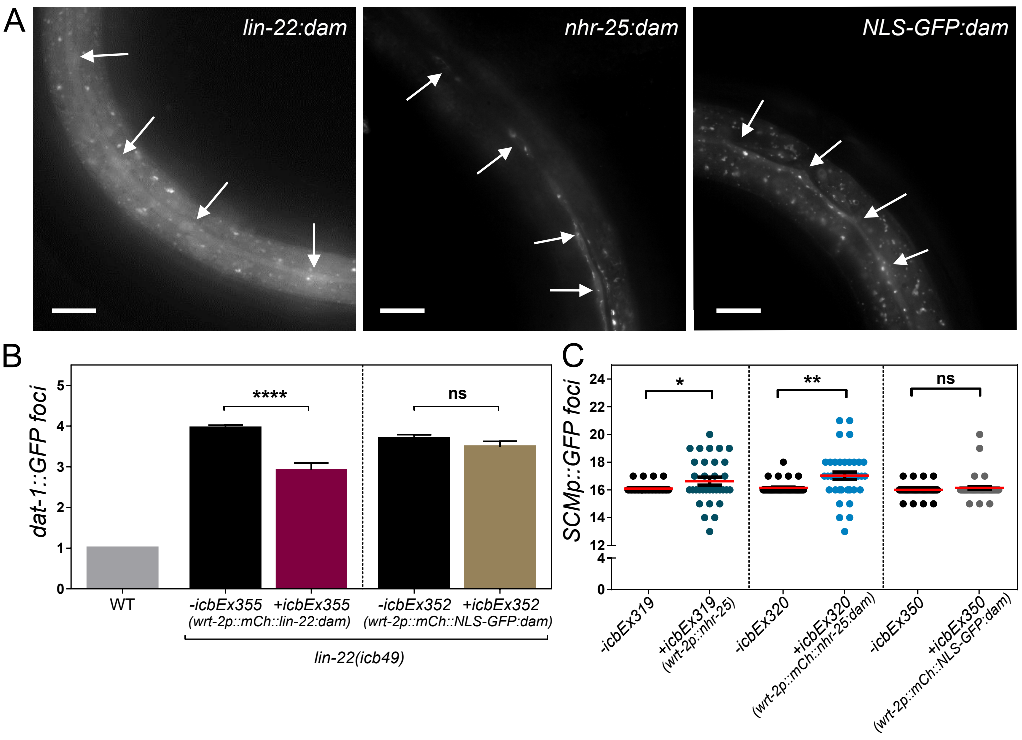

Figure S2: Validation of expression and functionality of TaDa transgenes.

(A) Confirmation of single-copy transgene expression in the *wrt-2* domain (seam cells) using *mCherry* expression (primary ORF). Animals were imaged at the L4 stage and white arrows mark weak expression in the seam, as expected for single copy transgenes. **(B)** Overexpression of the *lin-22:dam* TaDa construct as a multi-copy transgene (*icbEx355*) in the *lin-22(icb49)* mutant partially rescues the supernumerary PDE neurons phenotype labelled by *dat-1p::GFP*, whereas the overexpression of the control *NLS-GFP:dam* TaDa construct does not ($n \geq 33$). **(C)** Overexpression of *nhr-25* or the *nhr-25:dam* fusion from a TaDa construct as a multi-copy transgene under the *wrt-2* promoter increased the mean seam cell number, whereas the overexpression of the control *NLS-GFP:dam* TaDa construct did not ($n \geq 31$). Scale bars in A are

20 μm. In B and C error bars indicate standard error of the mean and black stars indicate statistically significant differences in the mean with a t-test (* $p<0.05$, ** $p<0.01$, **** $p<0.0001$).

Fig. S3.

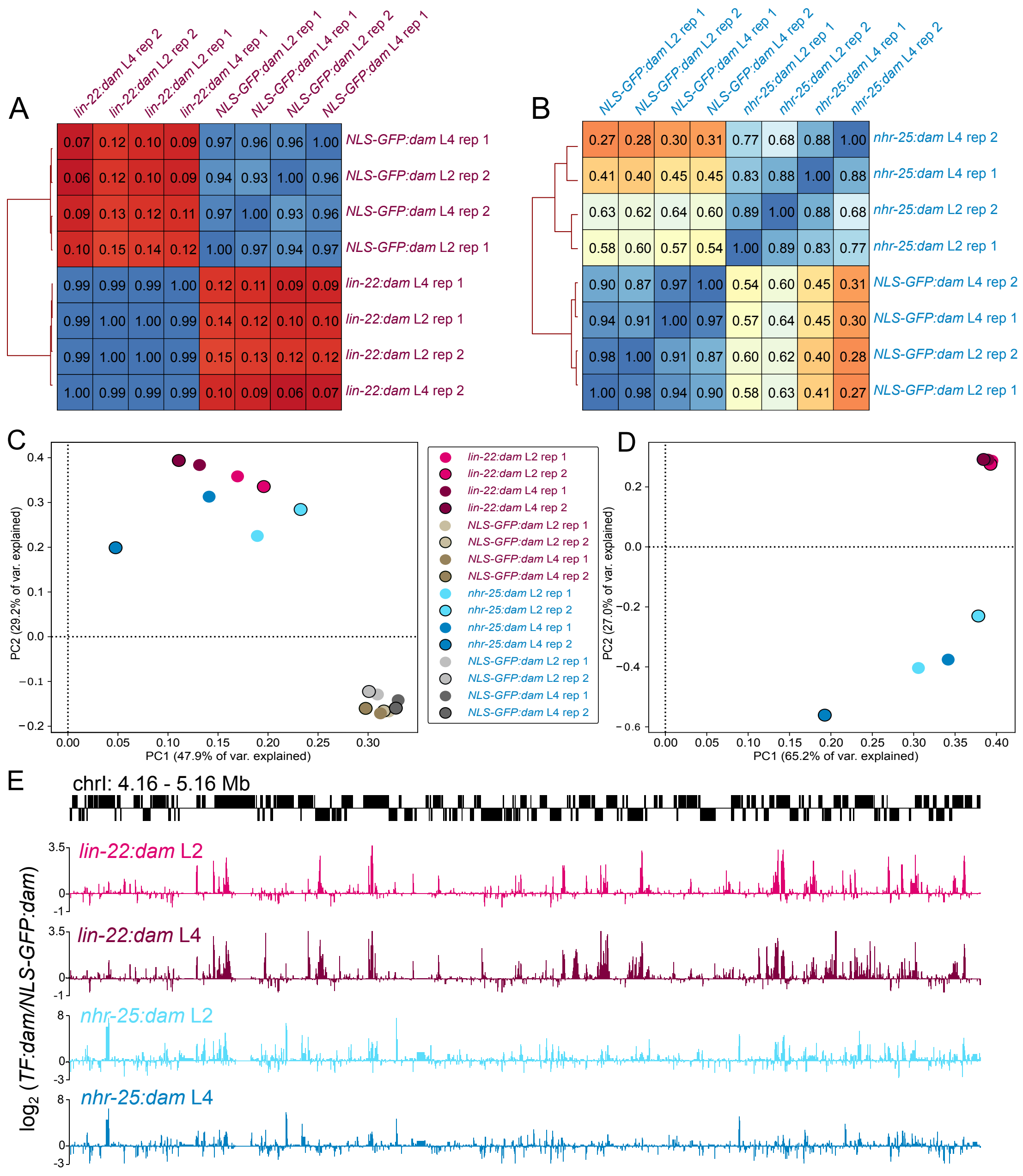

Figure S3: TaDa replicate reproducibility and fusion-dependent methylation.

(A-B) Pearson correlation heatmaps based on normalised aligned read count maps for the LIN-22 (A) and NHR-25 (B) TaDa experiments. The correlation coefficient for each pairwise comparison is printed in each cell of the heatmaps. All Dam-fusions show strong replicate reproducibility and

high within-fusion correlation, indicated by high correlation coefficients. Note that TF and control samples show low correlation between them and cluster separately. **(C)** Principal component analysis on normalised aligned read count maps for all samples shows distinct grouping between TF and control fusions. **(D)** Principal component analysis only on *lin-22:dam* and *nhr-25:dam* replicates shows distinct grouping between the two TF samples. Samples in C and D are colour-coded as per the shared key in the middle. **(E)** Example of averaged signal enrichment profiles for *lin-22:dam* and *nhr-25:dam* fusions across a 1 Mb region of chromosome I (locations of protein coding genes are indicated as black lines / boxes. Signal enrichment forms distinct peaks for each TF that show some similarity between stages. The Y-axes represent $\log_2$*(TF:dam/NLS-GFP:dam)* scores. Scale bar length is 2 Mb.

Fig. S4.

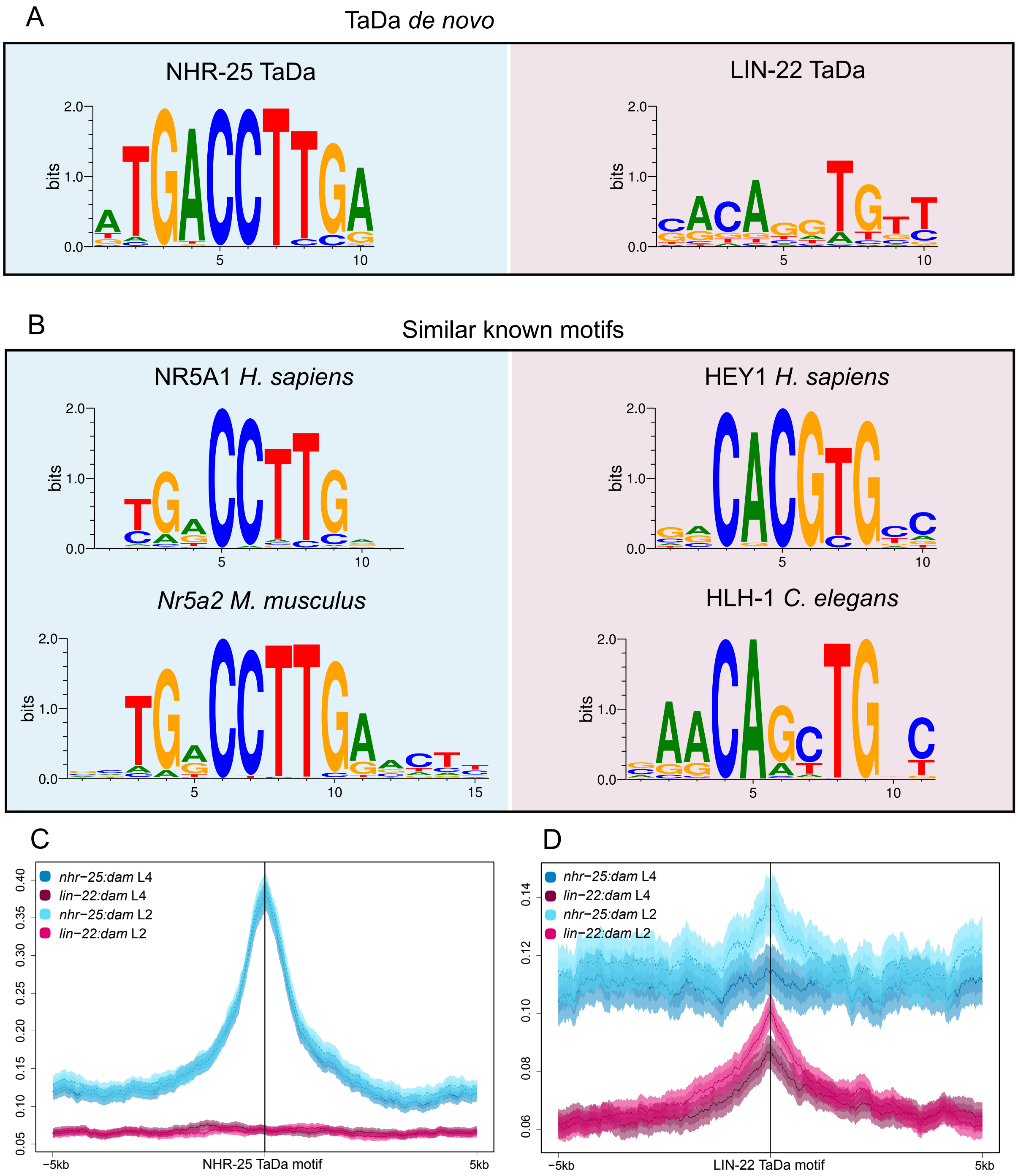

A
TaDa *de novo*
NHR-25 TaDa
LIN-22 TaDa
B
Similar known motifs
NR5A1 *H. sapiens*
HEY1 *H. sapiens*
Nr5a2 M. musculus
HLH-1 *C. elegans*
bits
C
D
nhr−25:dam L4
lin−22:dam L4
nhr−25:dam L2
lin−22:dam L2
−5kb
NHR-25 TaDa motif
LIN-22 TaDa motif
5kb

Figure S4: NHR-25 and LIN-22 binding motifs identified using TaDa peaks.

(A) *De novo* identified DNA motifs for NHR-25 (left) and LIN-22 (right) binding from TaDa peaks. These motifs were found in 41% of the LIN-22 L2, 33% of the LIN-22 L4, 47% of the NHR-25 L2 and 51% of the NHR-25 L4 total TaDa peaks, with the co-occurrence being statistically significant using Monte Carlo simulation (p-values < 0.0001 in all cases)**. (B)** Similar known motifs to the TaDa-identified NHR-25 (left) and LIN-22 (right) motifs are shown from available databases. **(C-D)** Aggregation plots of TaDa signal for all TFs and available stages over instances of the NHR-25 TaDa motif (C) and the LIN-22 TaDa motif (D). Both TFs show stronger preference for their respective motif compared to the alternative motif. Regions of ±5 kb around the peak centres have been plotted, the y-axes represent mean enrichment scores for the plotted sequences and shaded areas represent 95% confidence intervals.

Fig. S5

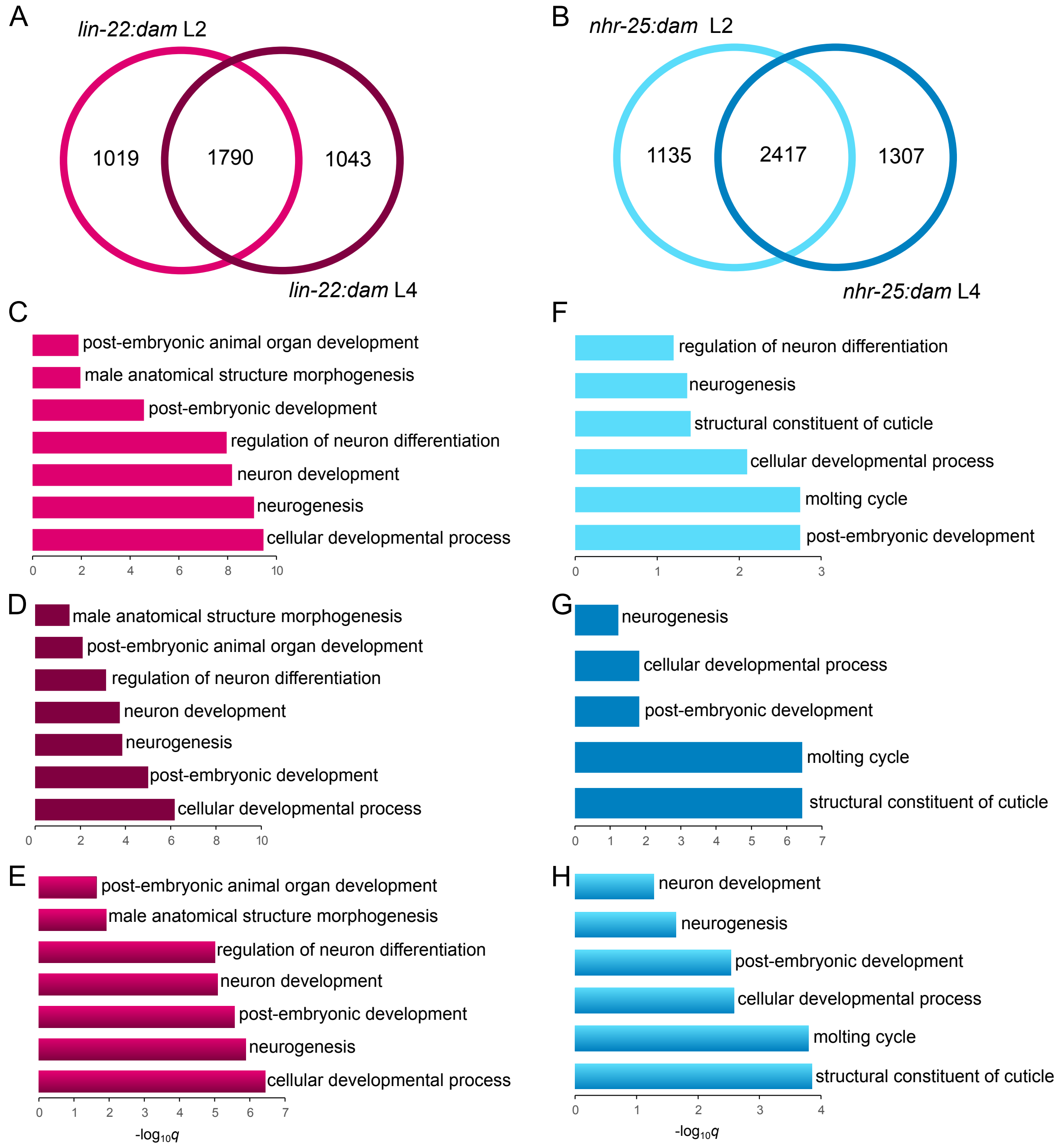

Figure S5: Enriched GO terms for LIN-22 and NHR-25 putative targets relate to their biological functions.

(A-B) Venn diagrams for putative target intersections between genes identified at L2 and L4 for the *lin-22:dam* (A) and *nhr-25:dam* fusions (B). The intersection in both cases is significant with a hypergeometric distribution test $p < 0.0001$). **(C-H)** Plots of selected significantly enriched terms from GO term analysis for the genes in the L2 (C,F), L4 (E,H) or intersection datasets (D,G). For both TFs at all stages, relevant terms are recovered related to neurogenesis, development or molting.

Fig. S6

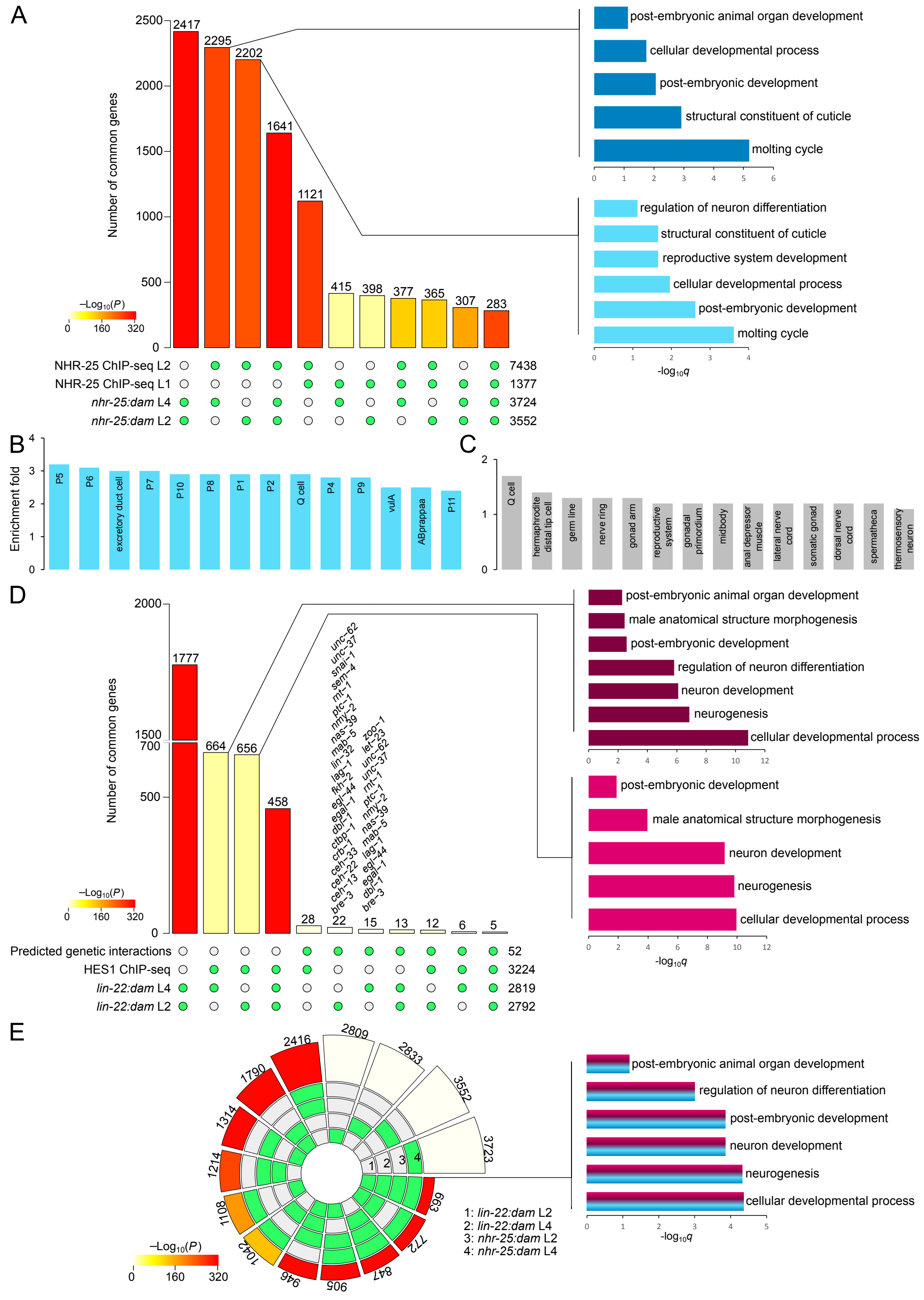

Figure S6: TaDa identified targets for NHR-25 and LIN-22 show significant overlaps between them and with other published datasets.

(A) Barplot showing the size of all possible intersections between: *nhr-25:dam* identified target genes at L2/L4, NHR-25 ChIP-seq-identified target genes at L1 (14) and NHR-25 ChIP-seq peaks at L2 (16) assigned to genes using the same method used in this study. Selected enriched GO terms for gene sets from the large intersections between TaDa and ChIP-seq L2 are shown. **(B-C)** Tissue-enrichment analysis in the gene set of putative targets that are common between NHR-25 L2 ChIP-seq and TaDa (B) or exclusively identified by ChIP-seq (C). In both cases, the top 15 most overrepresented tissues are shown. **(D)** Barplot showing the size of all possible intersections between: the identified target genes for *lin-22:dam* L2 and L4, genes predicted to be downstream genetic interactors of *lin-22* (Zhong and Sternberg, 2006) and the *C. elegans* orthologues of HES1 targets based on ChIP-seq (Encode project accession: ENCSR109ODF). Selected enriched GO terms for gene sets from the intersections between orthologues of HES1 ChIP-seq targets and TaDa targets are shown. Genes in the pairwise intersection between TaDa and predicted interactors are listed in full above the bars. **(E)** Plot showing all possible intersections between TaDa-identified target genes for LIN-22 and NHR-25 at L2 and L4. Selected enriched GO terms are shown for the genes common in all datasets. In A, D the size of each individual gene set is printed at the bottom right of each graph. In A, D and E the statistical significance of each intersection assessed by a Fisher's exact test is colour-coded as shown in the key (yellow, orange, red marks significant overlaps).

Fig. S7

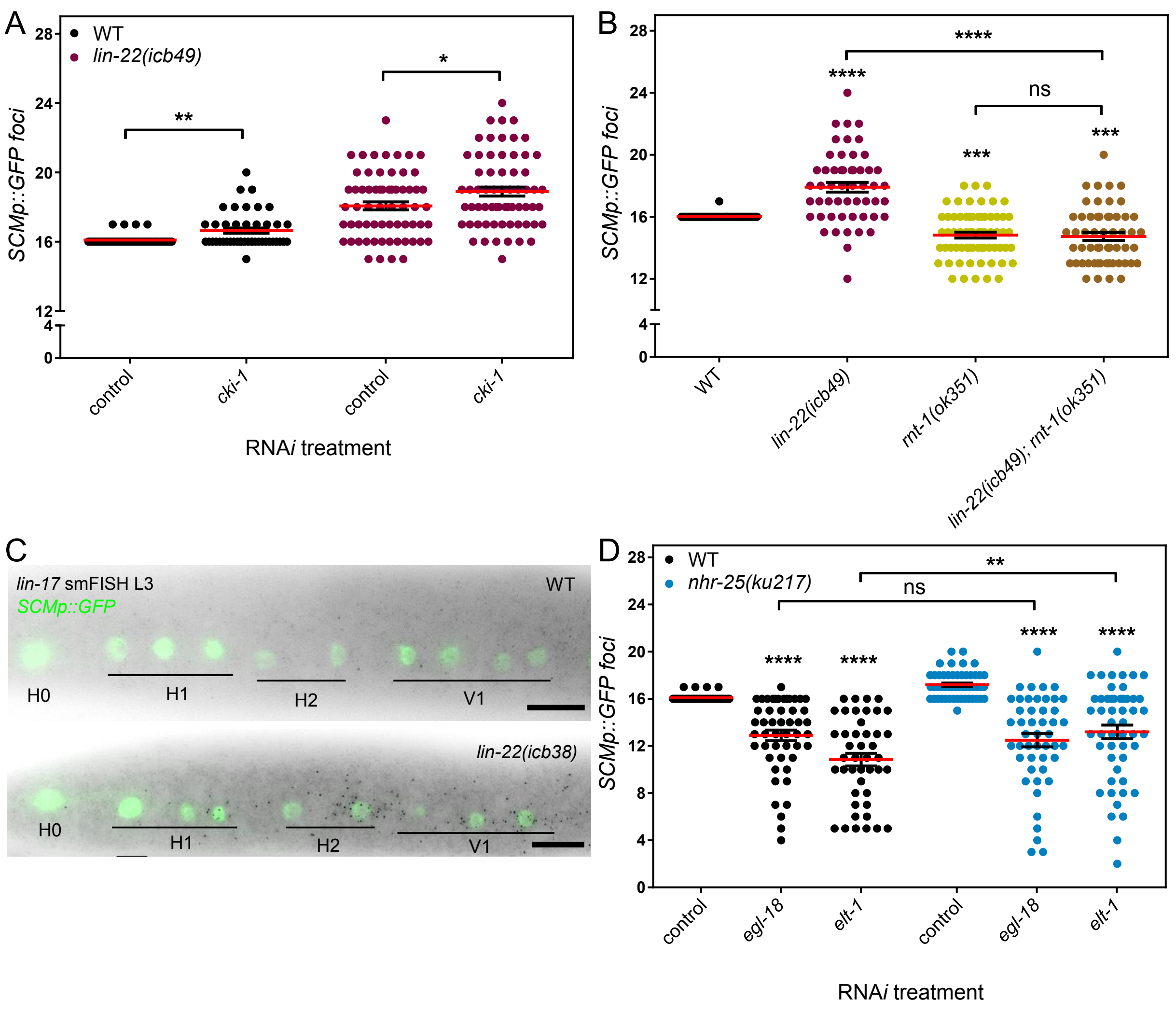

Figure S7: Genetic validation of regulatory relationships for TaDa-predicted targets of LIN-22 and NHR-25.

(A) Seam cell number scoring and comparison between wild-type and *lin-22(icb49)* mutants carrying the *SCMp::GFP* marker, treated either with control or *cki-1* RNAi (47≤n≤64). Knock-down of *cki-1* further increases the mean seam cell number of *lin-22* mutants. **(B)** Comparison of seam cell number between wild-type, *lin-22(icb49)* mutants, *rnt-1(ok351)* mutants and *lin-22(icb49); rnt-1(ok351)* double mutants carrying the *SCMp::GFP* marker (53≤n≤66). Note the

significant decrease in seam cell number in *lin-22(icb49); rnt-1(ok351)* double mutants, compared to the *lin-22(icb49)* single mutant phenotype, down to levels comparable to the single *rnt-1(ok351)* mutant. **(C)** Representative *lin-17* smFISH images from WT and *lin-22(icb38)* mutants showing increased *lin-17* expression in anterior seam cells (H1-V1) at the L3 stage. **(D)** Seam cell number scoring and comparison between wild-type and *nhr-25(ku217)* mutants carrying the *SCMp::GFP* marker, treated either with control, *egl-18* or *elt-1* RNAi ($44 \leq n \leq 53$). *egl-18* and *elt-1* knock-down in *nhr-25* mutants suppresses the increased seam cell number mutant phenotype. In C seam cells are labelled with *SCMp:GFP* and black spots correspond to *lin-17* mRNAs. The scale bars are 10 µm. In A, B and D error bars indicate standard error of the mean. Black stars indicate statistically significant differences in the mean with a t-test in A and a one-way ANOVA with a Sidak's multiple comparison test in B and D, * $p<0.05$, ** $p<0.01$, *** $p<0.001$, **** $p<0.0001$.

Fig. S8

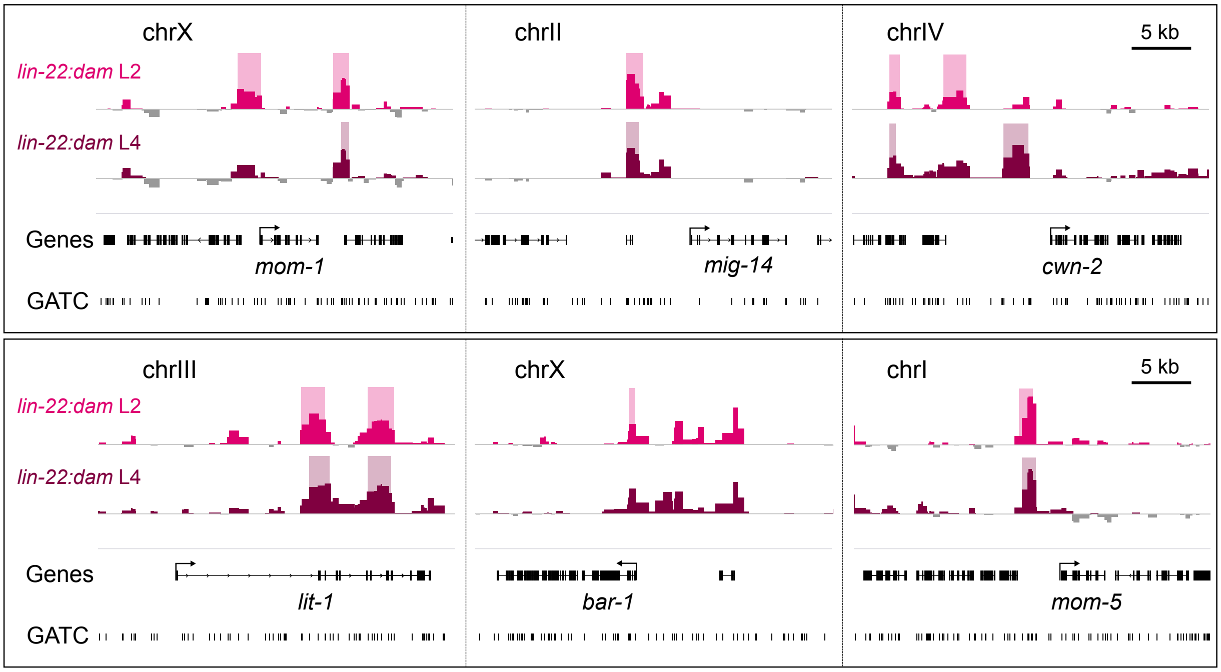

Figure S8: TaDa reveals a link between LIN-22 and multiple components of the Wnt signaling pathway.

Signal profiles from *lin-22:dam* showing enrichment forming statistically significant peaks (shaded regions) on sequences associating with the Wnt component-encoding genes *mom-1, mig-14, cwn-2, lit-1, bar-1* and *mom-5.* Y-axes are $\log_2$*(lin-22:dam/NLS-GFP:dam)* scores with data range: -1 – 3.5.

Separate files

Table S1. Genome coordinates of significant TaDa peaks for NHR-25 and LIN-22 at the L2 and L4 stage.

Table S2. Assigned genes per TaDa peak for NHR-25 and LIN-22 at the L2 and L4 stage.

Table S3. Strains used in this study.

Table S4. Primers used in this study (general oligos and smFISH probes).

Table S5. List of transgenes and injection mixes used in this study.
